# *Botrytis cinerea* causes different plant responses in grape (*Vitis vinifera*) berries during noble and grey rot: diverse metabolism versus simple defence

**DOI:** 10.3389/fpls.2024.1433161

**Published:** 2024-08-06

**Authors:** Kálmán Z. Váczy, Margot Otto, Adrienn Gomba-Tóth, Adrienn Geiger, Richárd Golen, Júlia Hegyi-Kaló, Thomas Cels, József Geml, Zsolt Zsófi, Ádám István Hegyi

**Affiliations:** ^1^ Food and Wine Research Institute, Eszterházy Károly Catholic University, Eger, Hungary; ^2^ Centre for Epidemic Response and Innovation (CERI), Stellenbosch University, Stellenbosch, South Africa; ^3^ HUN-REN-EKKE Lendület Environmental Microbiome Research Group, Eszterházy Károly Catholic University, Eger, Hungary; ^4^ Institute for Viticulture and Enology, Eszterházy Károly Catholic University, Eger, Hungary

**Keywords:** *Vitis vinifera*, *Botrytis cinerea*, noble rot, grey rot, metatranscriptomics, plant-pathogen interaction

## Abstract

The complexity of the interaction between the necrotrophic pathogen *Botrytis cinerea* and grape berries (*Vitis vinifera* spp.) can result in the formation of either the preferred noble rot (NR) or the loss-making grey rot (GR), depending on the prevailing climatic conditions. In this study, we focus on the functional gene set of *V. vinifera* by performing multidimensional scaling followed by differential expression and enrichment analyses. The aim of this study is to identify the differences in gene expression between grape berries in the phases of grey rot, noble rot, and developing rot (DR, in its early stages) phases. The grapevine transcriptome at the NR phase was found to exhibit significant differences from that at the DR and GR stages, which displayed strong similarities. Similarly, several plant defence-related pathways, including plant-pathogen interactions as hypersensitive plant responses were found to be enriched. The results of the analyses identified a potential plant stress response pathway (SGT1 activated hypersensitive response) that was found to be upregulated in the GR berry but downregulated in the NR berry. The study revealed a decrease in defence-related in *V. vinifera* genes during the NR stages, with a high degree of variability in functions, particularly in enriched pathways. This indicates that the plant is not actively defending itself against *Botrytis cinerea*, which is otherwise present on its surface with high biomass. This discrepancy underscores the notion that during the NR phase, the grapevine and the pathogenic fungi interact in a state of equilibrium. Conversely the initial stages of botrytis infection manifest as a virulent fungus-plant interaction, irrespective of whether the outcome is grey or noble rot.

## 1 Introduction

Noble rot (NR) is caused by a rare *B. cinerea* infection of grape berries (*Vitis vinifera*) under specific microclimatic conditions, including humid nights, foggy mornings, and dry sunny days (Ribéreau-Gayon et al., 1980; Magyar, 2011; Vannini and Chilosi, 2013). This phenomenon is utilised to produce world-renowned wines such as the *Sauternes AOC* and *Tokaji Aszú* in France and Hungary respectively (Ribéreau-Gayon et al., 2006; Teissedre and Donèche, 2013). However, under conditions of continuous mild wet weather, which are more common, this necrotrophic pathogen can cause grey rot (GR) that causes significant economic losses for growers worldwide (Williamson et al., 2007).

During the development of NR (developing rot, DR), two distinct phenotypical changes occur in grape berries. Initially the berry undergoes a colour change from green and yellow to purple-brown. Subsequently physical changes in the berry skin manifest as cracks in the cuticle layers leading to dehydration and an increase in sugar concentration (Magyar, 2006). Furthermore, additional biochemical changes take place including an increase in sugar alcohols and the formation of aroma precursors and volatile thiols (Tominaga et al., 2006; Thibon et al., 2011). These contribute to the distinctive aroma profile of NR wines (Magyar, 2006). Conversely, unfavourable weather conditions, such as excessive precipitation and a lack of dry periods, can lead to the develop grey rot instead of noble rot, resulting in the loss of the berry. This can have a detrimental impact on wine quality by interfering with the fermentation process negatively altering the sensory characteristics of the product (Hornsey, 2007; Morales-Valle et al., 2011).

In addition to the significant impact of microclimate on the development of NR and GR respectively, the latter is largely attributed to the intricate interaction dynamics that occur between *B. cinerea* and *V. vinifera* (Lovato et al., 2019; Haile et al., 2020). Only a limited number of studies have examined the differences in the gene expression profile of *V. vinifera* in response to *B. cinerea* infection during the onset of NR and GR respectively. Blanco-Ulate et al. (2015) examined the alternation in the expression profile of the *B. cinerea* and *V. vinifera* cv Sémillon gene during three distinct phases of NR, with a particular emphasis on berry development and metabolism, while Lovato et al. (2019) conducted a comprehensive analysis of NR development in two grapevine cultivars (Garganega & Müller-Thurgau) to identify both common and specific grapevine responses during botrytization. Moreover, these data were compared with transcriptome data generated from six grapevine cultivars exhibiting symptoms of postharvest wilting (Zenoni et al., 2016) and two cultivars displaying symptoms of grey rot GR (cultivars Tricadeira (Agudelo-Romero et al., 2015) and Marselan (Kelloniemi et al., 2015)). This study identified key genes that are modulated during NR were identified. Similarly, more recently Pogány et al. (2022) recently compared the transcriptome expression profile of *V. vinifera* in terms of redox and hormone-related genes between NR and GR. In a further study, Haile et al. (2017, 2020) examined the postharvest changes in the gene expression profile of the latter species during the quiescent phase (healthy infected berries) and the pre-egress phase (ripe grape berries devoid of any discernible *B. cinerea* infection) and egress phase (ripe grape berries exhibiting visible growth of *B. cinerea)*, which is illustrative of GR. The findings of this study indicate the plant defence responses impede the initiation of grey rot (GR) by *Botrytis cinerea*. Conversely the ripening associated fruit cell wall self-disassembly, coupled with elevated humidity facilitates the development of GR. Infection of grapes by *B. cinerea* under optimal conditions results in the onset of the noble rot process. Most experts and researchers concur that this process can potentially take an unfavourable turn at any time, resulting in yield loss, which phenomenon is called grey rot (Williamson et al., 2007). The objective of this study is to ascertain the relationship between the transcriptome profile of *Vitis vinifera* genes in early noble or grey rot, i.e. DR berry type, and the NR and GR rot profiles. The objective is to determine whether the process of noble rot can turn into grey rot in the case of unfavourable changes in external conditions or whether the DR stage is more like grey rot but, if favourable weather conditions prevail over an extended period, the noble rot berry stage may emerge. Given the observable distinction between NR and GR berries in terms of their physical and chemical characteristics, we hypothesise that the formation of these two types of berries is subject to different plant regulatory processes. These implies the existence of functional difference between the expressed *Vitis vinifera* genes. The hypothesis that the resulting NR berry will remain stable under favourable retaining its physical and chemical properties and nutritional quality over a longer period suggests a further functional difference. Additionally, it is also hypothesised that during the NR phase, the plant will exhibit a reduced response to plant pathogens and other metabolic processes will be active, despite the active and abundant presence of *Botrytis cinerea*.

## 2 Materials and methods

### 2.1 Sampling, RNA extraction and data analysis

The sampling was conducted in the ‘Betsek’ vineyard located in the village of Mád in the Tokaj wine region of Hungary. The grape berries of cv. *Furmint* were randomly selected in replicates of five from healthy (H, or phase I), developing rot (DR, or phases II and III) noble rot (NR, or phase IV) and grey rot (GR or phase VI) berry types during September (S), October (O), and November (N) in 2017, respectively. This resulted in a total of 60 samples. The different types of berries were classified based on visual and textural parameters (Naegele, 2018; Hegyi-Kaló et al., 2020) as follows: i) healthy berries are whole green or yellow green berries without any lesion spots with a completely healthy stem joint, ii) phase II developing rot berries with brown of light brown lesion spots, iii) phase III developing rot berries are almost completely brown coloured, moderately shrivelled iv) noble rot berries are brown or purple brown, completely shrivelled with a moderate to high amount of fungal mycelia and having soft, fleshy texture and v) grey rot berries are different in colour from NR, more brown or light brown with a dry, hard, or completely degraded texture with advanced berry skin degradation, sometimes leaking onto the skin. The collected berries were stored aseptically in 50 ml tubes which were placed in liquid nitrogen until RNA extraction was performed in accordance with the methodologies outlined by Reid et al. (2006); Hegyi et al. (2022) and Otto et al. (2022). Briefly, the lysis was initiated by adding 5 g of powdered sample to 20 ml of extraction buffer [300 mM Tris HCl (pH 8.0), 25 mM EDTA, 2M NaCl, 2% CTAB, 2% PVPP, 0.05% spermidine trihydrochloride] followed by incubation. The extraction was performed by adding an equal volume of isoamyl alcohol (24:1) followed by two washing steps of the resulting supernatant by centrifugation. The precipitation of RNA was achieved by centrifugation with the addition of the tenth of the volume of 3M sodium acetate buffer (pH = 5.2) resulting in a formation of a pellet that was lyophilised. Subsequently, the lyophilised pellet was then dissolved in 1 ml of TE buffer [300 mM Tris HCl (pH=8), 25mM EDTA] to which 8M LiCl was added. Following an overnight incubation period, a final washing step was performed using 70% ice-cold ethanol and centrifugation. This resulted in the formation of a precipitate which was dried using a lyophilizer. Finally, 42 µl of nuclease-free water and 2% mercaptoethanol were added to the lyophilised RNA, after which its quality was assessed using a Nanodrop 2000 instrument (Thermo Fischer Scientific, Waltham, Massachusetts, USA). The extracted RNA was stored at -20°C until sequencing. The sequencing was then performed on the Illumina NextSeq500 platform at the UD GenoMed Medical Genomic Institute of the University of Debrecen, yielding 7.8-40.6 million single-ended reads per sample with an average length ranging between 70-75bp.

A quality check of the sequenced reads was conducted using the FastQC v0.11.5 software tool (Babraham Bioinformatics, Cambridge, UK). The reads were trimmed and filtered using the FASTX-TOOLKIT (http://hannolab.cshl.edu/fastx_toolkit/index.html). The resulting high-quality reads were normalised in accordance with the methodology described by Howe et al. (2014) and Pell et al. (2012) using the Khmer v2.1.1 and then aligned with the *Vitis vinifera* X12 genome (BioProject: PRJEA18785) with Salmon v1.3.0 in order to generate normalised TPM abundance counts. The gene names were linked to their respective GTF annotations using the *GenomicFeatures* package in R. All *Vitis vinifera* RNA sequences were uploaded to the NCBI database under the BioProject PRJNA736205 and the BioSample SAMN19612984.

### 2.2 Statistical analyses

A statistical analysis was conducted on the combined *Vitis vinifera* and *Botrytis cinerea* functional gene set obtained from all phases [H (Phase I), DR (Phases II and III), NR (Phase IV) and GR (Phase VI)] and date (September, October and November) using the R software tool (R Core Team, 2023). To ascertain how the dependent variables of richness (number of transcript types present), and abundance (number of mapped transcript reads) changed with respect to the date and grape berry phase, several ANOVA models were evaluated. The Tukey’s test was used to determine significant pairwise differences between the sample types. The aforementioned test revealed that the grape berry phase was the most effective at describing the data, thus a one-way ANOVA was conducted on the respective phases. Furthermore, nonmetric scaling (NMDS) analyses were performed on the median of the ratios of the abundance counts of *V. vinifera* to obtain a visual representation of the changes in composition across the respective phases and dates. To this end, the data were subjected to 999 iterations per run using the Bray-Curtis index (Sørensen similarity), commencing with a random number. Furthermore, a nonparametric multivariate statistical permutation test (PERMANOVA) was performed with 9999 permutations to determine whether the functional genes of *V. vinifera* exhibited statistical differences according to the phase and date of the grape berry rot. The differential gene expression analyses were generated using the DeSEQ2 package version 1.42 of R programming (Love et al., 2014) and the pathway enrichment analyses were performed with the online tool for KOBAS enrichment analyses online tool (Xie et al., 2011). The annotated functional genes then transcribed to proteins, from which functional analyses were performed using the STRING core data resource (Szklarczyk et al., 2023).

## 3 Results

### 3.1 Quantitative analyses of *V. vinifera* gene set

**Figure 1:**
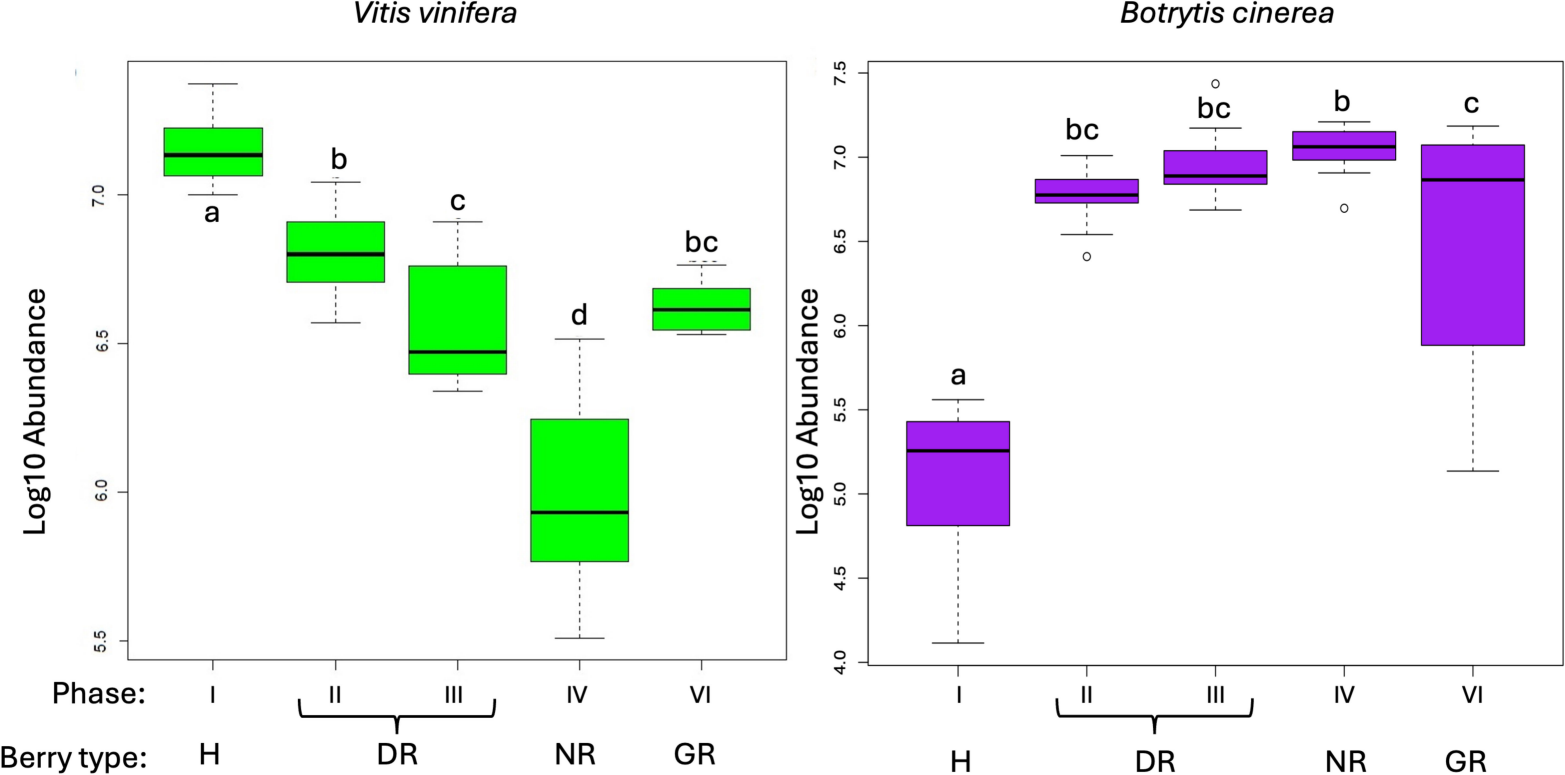
Box plot of the abundance distribution of *Vitis vinifera* (left) and *Botrytis cinerea* (right) transcripts for all berry types. Phases and berry or rot types are indicated on the horizontal axis. Abundance values are plotted using a decimal logarithmic transformation. Lowercase letters indicate significant differences according to the Tukey HSD test.

The sum of abundance of annotated *Vitis vinifera* and *Botrytis cinerea* genes was compared between sample types using an ANOVA test to identify differences between the phases of noble rot and grey rot. **Figure 1** shows the box plot of the total abundance distributions of the various berry types. In the case of *Vitis vinifera*, the highest total abundance was observed in the healthy berry, followed by phases II and III, which together are referred as the DR stage. The NR phase exhibits a significantly reduced abundance than this, while the GR phase shows a distribution that is not statistically different from phases II and III, which collectively corresponds to the DR type. In contrast to the grapevine, the opposite trend is observed in botrytis. The total abundance is lowest for healthy berries and significantly higher for DR. The NR phase shows a higher mean value, but this difference is not significant according to the ANOVA test. The GR berry type shows the same abundance distribution as the DR type.

A quantitative analysis of expressed genes revealed that the highest total abundance was observed in the healthy berry, while the lowest abundance was found in NR. The total abundance of the remaining berry types fell between the forementioned two values, with DR and GR exhibiting approximately similar values. In this context, the term “total abundance” refers to the number of reads sequenced from the samples. This result indicates that the grapevine is now in a less metabolically active state, that the NR process is complete, and the plant shows a metabolic slowdown and greatly reduced activity. However, as will be discussed, the results of a functional analysis of grapevine genes lead to the opposite conclusion.

**Figure 2:**
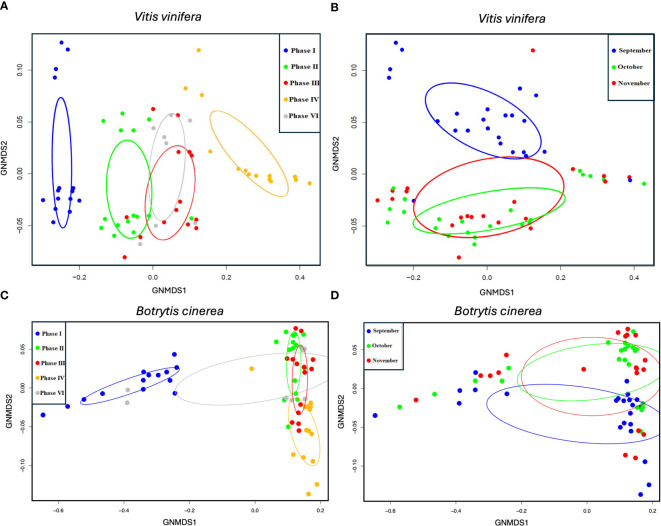
NMDS ordination plots for genes annotated with *Vitis vinifera* **(A, B)** and *Botrytis cinerea* **(C, D)**. Different colouring of the samples is used to show separations by berry types **(A, C)** and sampling month **(B, D)**.

NMDS analyses revealed a pronounced and statistically significant separation between the functional gen sets of *V. vinifera* and *Botrytis cinerea* with respect to phase types (**Figure 2**.). This finding was confirmed by the PERMANOVA statistical test, which demonstrated a significant correlation between the functional genes of *V. vinifera* and the proportions of the variation for grape berry phases (*p* < 0.001***, r^2^ = 0.36). Similarly, the PERMANOVA test indicated a notable for berry phases in the case of *Botrytis cinerea* (p < 0.001***, r^2^ = 0.67). The examination of the separation by sampling dates revealed no significant differences in either case.

The gene set of the healthy berry is completely distinctive, as is the gene set of the NR berry. However, the gene sets of the GR and DR phases exhibit partial overlap. For purposes of comparison, the group colouring by sampling month has also been plotted, which shows less difference and also similar ordinations of the identified *Botrytis cinerea* genes. In the case of grapevine genes, the GR and DR phases do not show a clear, however in the context of *Botrytis* genes, the GR samples are shared between healthy, DR and NR samples. The slight separation of the functional gene expression abundance profile of V. vinifera in terms of date in the NMDS analyses in the current study is also in accordance with the findings of Pogány et al. (2022). This indicates the influence of the date on both NR and GR development, but this is on selected gene types, i.e., those highlighted by the enrichment analyses in the current study.

### 3.2 Functional analyses of *V. vinifera* gene set

**Figure 3:**
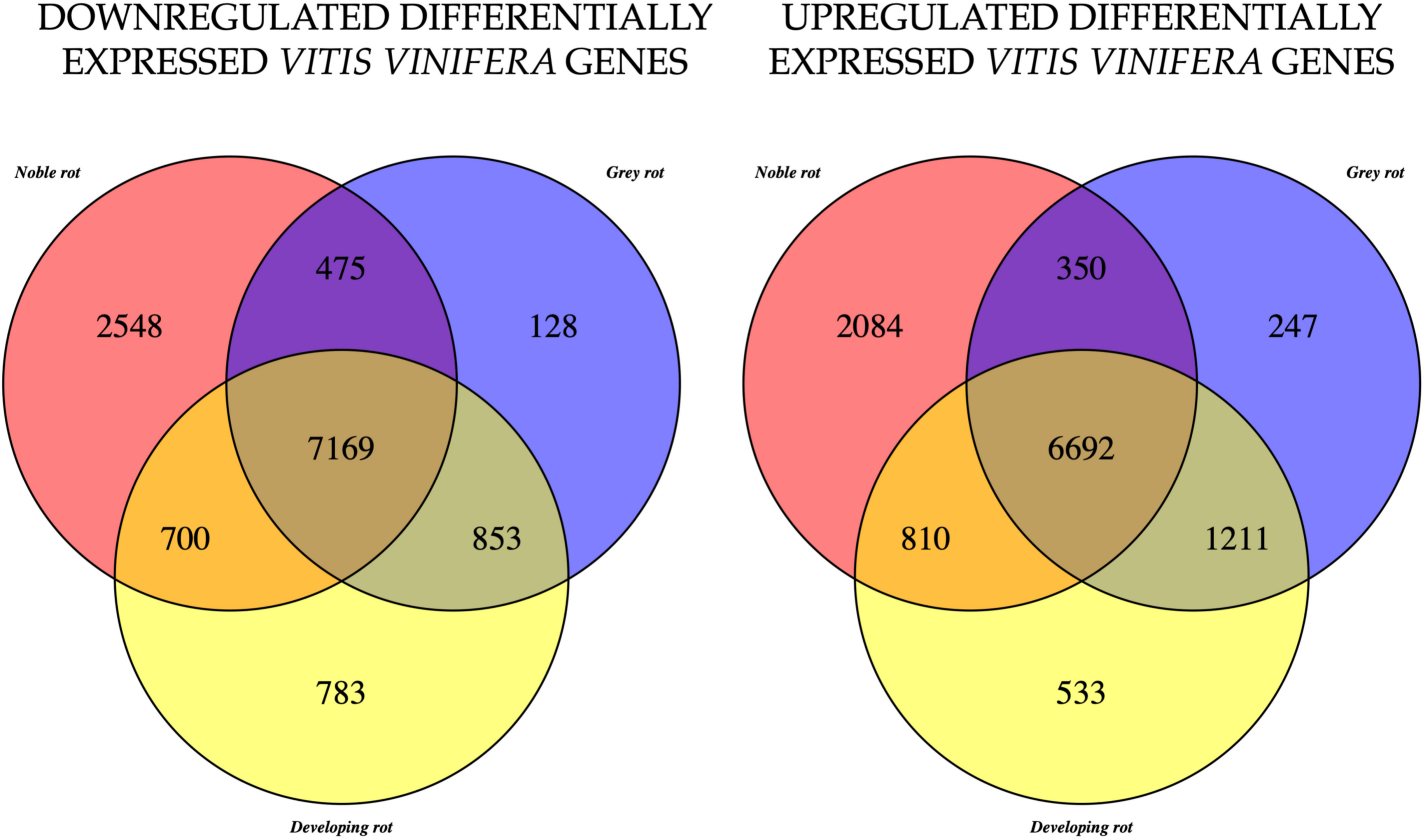
Venn diagram, of the down-regulated (left) and up-regulated (right) *Vitis vinifera* genes, in the case of noble rot (NR), developing rot (DR) and grey rot (GR). Differential expression analyses were performed against the healthy berry gene set.

The remaining analyses will focus on the functional genes of grapevine. Subsequent analyses were conducted on the samples according to rot types, i.e. healthy (H), developing rot (DR), noble rot (NR) and grey rot (GR). The objective was to ascertain which genes are differentially expressed (DEG) in the other berry types in comparison to the expression levels of the functional genes that can be determined in healthy berries. The results are summarised in **Figure 3**. The majority of the genes that were either up- or downregulated were identified in the common set of the three berry types. However, a significant number of genes were also identified as differentially expressed in the other sets. The figure illustrates that the DR type has a greater number of DEGs in common with GR than with NR in both in the up- and downregulated cases (1211 vs. 810 and 853 vs. 700 respectively). Notwithstanding the lowest total abundance, the number of uniquely expressed genes in NR that are up- or down-regulated is considerable (2084 and 2548 respectively).

We examined which genes exhibited increased expression in GR and decreased expression in NR and vice versa. A subsequent filtering for the NR upregulated and GR downregulated genes yielded a mere two genes in contrast to the 452 genes identified in the reverse comparison. Based on the findings, we have examined which pathways are upregulated in GR and downregulated in NR. Some of these are pathways that are significantly upregulated in the H vs GR comparison, as indicated in **Table 1**. Moreover, we identified significantly enriched pathways that were not included in the results of previous analyses.

**Table 1:** The enriched *Vitis vinifera* metabolic pathways for noble rot (NR), grey rot (GR) and developing rot (DR) berry types.

KEGG term	Enrich ratio		
	NR	GR	DR
**Metabolic pathways**	0.019	0.032	0.033
**Biosynthesis of secondary metabolites**	0.019	0.032	0.035
Phenylalanine, tyrosine and tryptophane biosynthesis	0.04		
**Biosynthesis of amino acids**	0.022	0.027	0.035
Valine, leucine, and isoleucine biosynthesis	0.059		
Pantothenate and CoA biosynthesis	0.038		
Valine, leucine, and isoleucine degradation	0.036		
2-Oxocarboxylic acid metabolism	0.016		
One carbon pool by folate	0.050		
Folate biosynthesis	0.037		0.037
Cutin, suberine and wax biosynthesis	0.036		
Peroxisome	0.025		
Propanoate metabolism	0.045		
Pyruvate metabolism	0.011	0.034	
Stilbenoid, diarylheptanoid and gingerol biosynthesis	0.051		
Flavonoid biosynthesis	0.009		
RNA polymerase	0.174		
Galactose metabolism	0.071		
Other types of O-glycan biosynthesis	0.067		
Tryptophan metabolism	0.059		
Sesquiterpenoid and triterpenoid biosynthesis	0.058		
Tyrosine metabolism		0.111	0.111
Tropane, piperidine and pyridine alkaloid biosynthesis		0.105	0.105
Isoquinoline alkaloid biosynthesis		0.103	0.103
**Alanine, aspartate and glutamate metabolism**		0.087	0.087
Arginine and proline metabolism		0.065	0.065
alpha-Linolenic adic metabolism		0.109	0.094
Fatty acid degradation		0.061	0.061
**Glycolisis**		0.047	0.047
Ether lipid metabolism		0.091	0.091
Inositol phosphate metabolism		0.052	0.052
Glycerophsholipid metabolism		0.031	0.041
Carbon fixation in photosynthetic organisms		0.077	0.077
**Carbon metabolism**		0.03	0.034
Aminos sugar and nucleotid sugar metabolism		0.034	0.041
**Fructose and mannose metabolism**		0.034	
Butanoate metabolism		0.036	
beta-Alanine metabolism		0.035	
Taurine and hypotaurine metabolism		0.417	0.5
Isoflavonoid biosythesis		0.286	0.286
Vitamin B6 metabolism		0.2	0.267
Betalain biosynthesis		0.167	0.167
**Nicotinate and nicotinamide metabolism**		0.125	0.125
Cysteine and methionine metabolism			0.064
**Glycine, serine, and threonine metabolism**			0.045
Riboflavin metabolism			0.063
Arachidonic acid metabolism			0.1

Pathways forming gene clusters are indicated with the same colour. The colouring used for different berry types does not mean that the pathways belong to the same cluster, they have different meaning for each berry types. Enrichment ratios are indicated for rot types. The bold terms indicate processes that are up-regulated in NR and down-regulated in GR.

To functionally determine different gene sets, we performed KOBAS enrichment analyses on the gene sets that were upregulated identified for the types of NR, GR, and DR berries to determine significantly enriched pathways. The enrichment ratios for the significant Kegg pathways (Kanehisa et al., 2017) for each type of rot are shown in **Table 1**.

**Figure 4:**
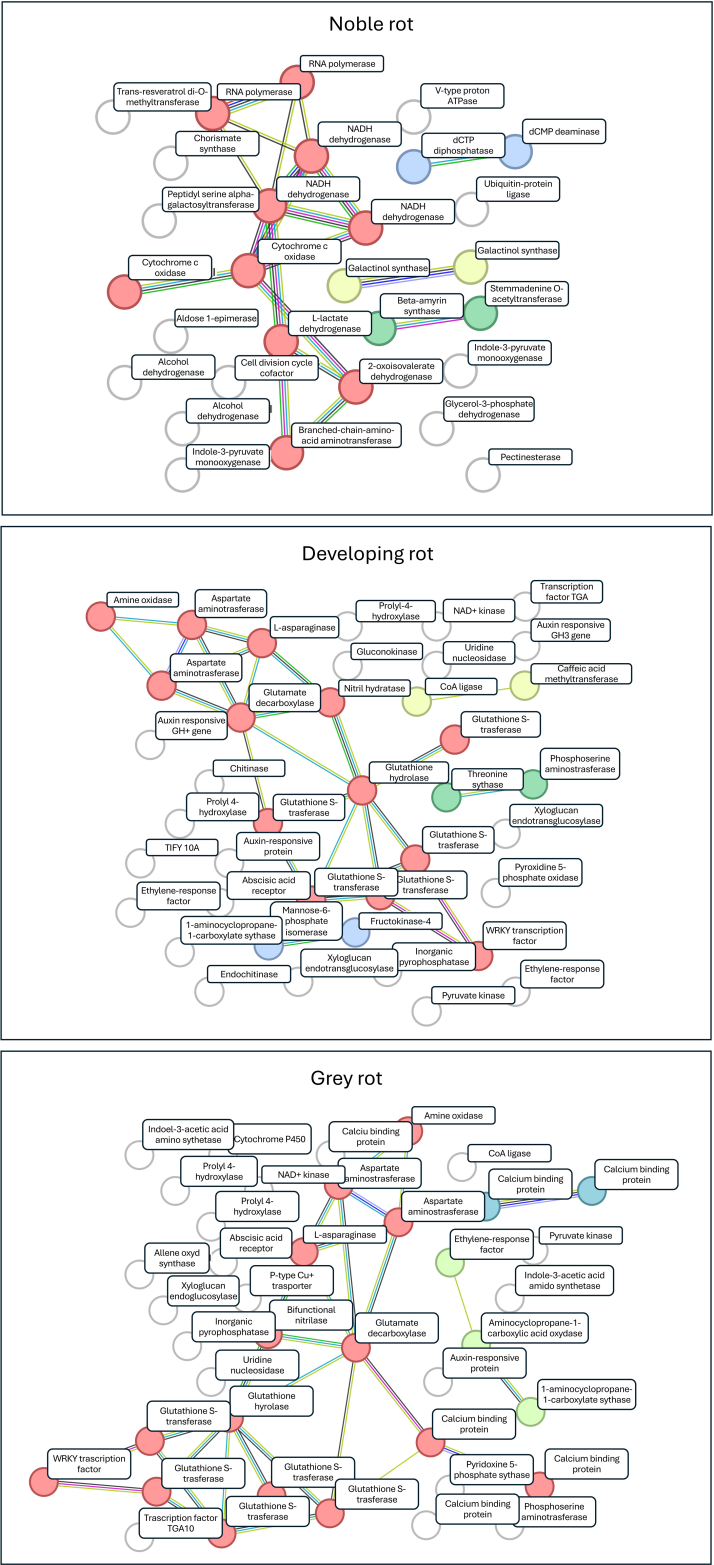
Core clusters for the gene sets of NR, GR and DR transcriptomes. The k-mean clustering modules are indicated by circles; gene names are indicated using KEGG nomenclature (find UniprotKB IDs in **Supplementary Table 4**).

The upregulated *Vitis vinifera* genes were cross-referenced with Uniprot protein IDs (Boutet et al., 2007), and subsequently analysed using the STIRNG platform for the NR, GR, and DR datasets. For each type of the dataset were clustered using k-mean clustering with the minimum cluster number, as determined by the algorithm set at k=4 for each rot type. The Protein-Protein Interaction (PPI) enrichment p-value (PPI) was 1.63*10^-4^, 1*10^-16^ and 1.11*10^-16^, respectively for NR, GR, and DR respectively, indicating that the networks have higher number of interactions than would be expected by chance. The results of the gene ontology functional enrichment for the biological process and molecular function of each type of berry are presented in **Supplementary Tables 1–3**. The k-mean clustered functional networks for NR, GR and DR are illustrated in **Figure 4**, with the gene names identified from the KEGG database provided as annotations. Gene names are transcribed to UniprotKB protein IDs which are listed in **Supplementary Table 4**.

It is of particular interest to highlight the results pertaining to functional pathways that are up-regulated in GR but down-regulated in NR. Some of these pathways are significantly enriched in the GR vs. H comparison, including alanine, aspartate and glutamate metabolism (enrichment ratios: 0.065), Glycolysis (0.079), Carbon metabolism (0.049), Fructose and mannose metabolism (0.085), Nicotinate and nicotinamide metabolism (0.25) and Glycine, serine and threonine metabolism (0.058). Additionally, we identified pathways that were not included in the results but were found to be upregulated in GR and downregulated in NR. These pathways are the Pentose phosphate pathway (enrichment ratio: 0.082), Phenylalanine metabolism (0.073), Sulphur relay system (0.143), Amino sugar and nucleotide sugar metabolism (0.041) and Plant-pathogen interaction (0.034).

**Figure 5:**
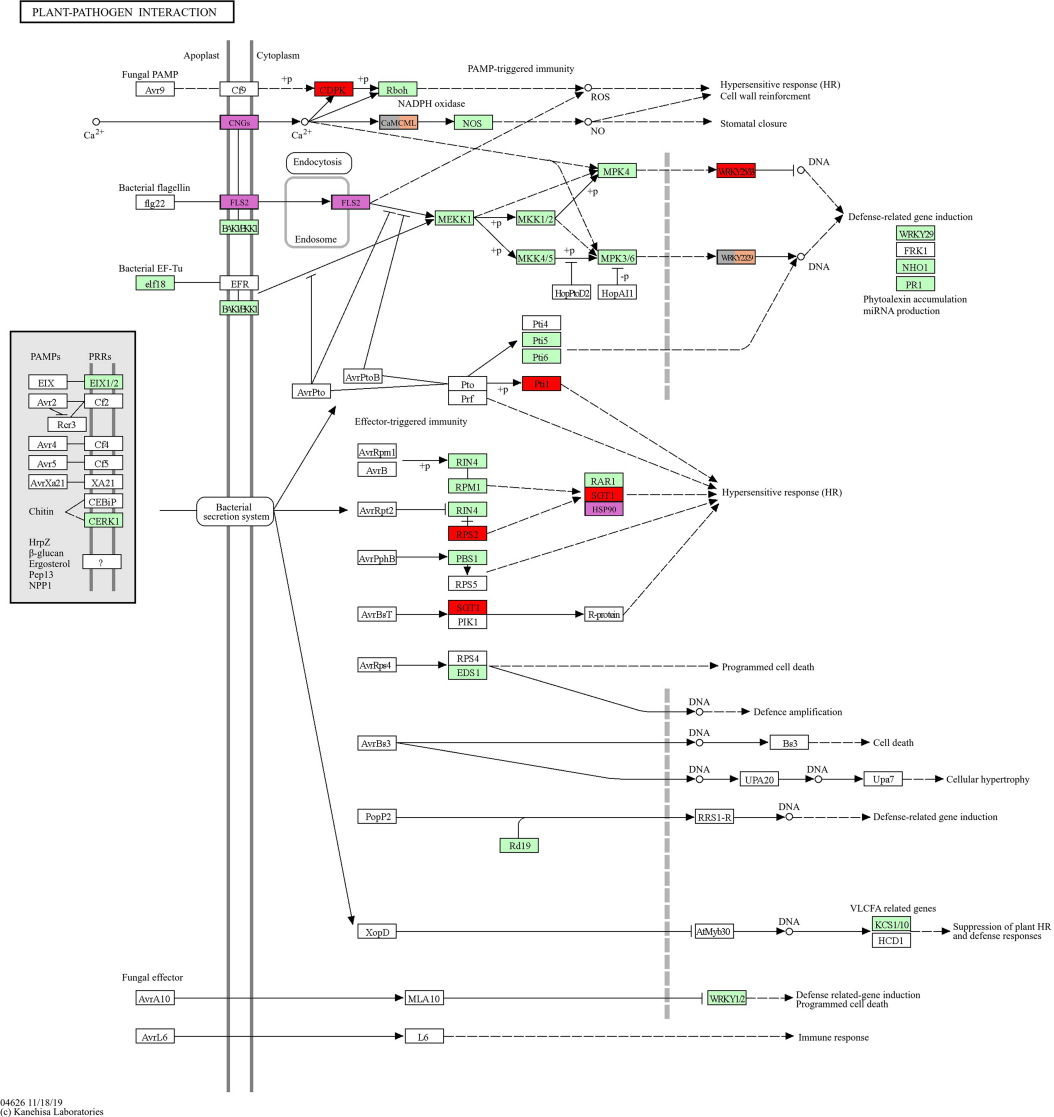
The figure shows a schematic diagram of the plant pathogen interaction pathway of grapevine (*Vitis vinifera*). The boxes in green are linked to entries via the conversion of k-numbers to gene identifiers within the reference pathway. This indicates the presence of genes within the genome and the completeness of the pathway. The boxes in red are those that are up-regulated in GR and down-regulated in NR. The boxes in purple are genes expressed up-regulated in NR, the orange boxes are genes up-regulated in DR, and the grey boxes are genes up-regulated in GR (up-regulated in GR but NOT down-regulated in NR). These last two groups contain the same two genes. The figure is based on the online KEGG pathway map (https://genome.jp/kegg-bin).

**Figure 5**, generated with the KEGG database pathway plotter tool (https://genome.jp), offers an immediate and straightforward depiction of the similarities and differences between the rot types affecting the plant-pathogen relationship. The results obtained for NR, GR, and DR are summarized, with particular emphasizes on the distinctions between NR and the other two types.

## 4 Discussion

Our study represents, the first comprehensive analyses of the *V. vinifera* gene expression profile from H grape berries during the various stages of NR and GR development in the field, over three harvest dates. Our findings reveal notable differences in the quantity and composition of expressed genes between these aforementioned phases as well as between different harvest times. The study offers a comprehensive and coherent overview of the *V. vinifera* defence-related genes throughout the progressive NR stages as well as GR. The objective of this study is to determine the distinctions between grey rot and noble rot in terms of the grapevine plant functions. The results demonstrate while the berry under GR conditions the grape predominantly expresses a response to pathogen infection, a diverse metabolic process occurs in the noble rot condition.

### 4.1 Decreased and separating NR gene set

The distribution of *Vitis vinifera* gene abundances in berry stages was compared with the abundance of *Botrytis cinerea* genes sequenced and annotated from samples. This is in contrasts to the increase in the abundance and richness of the *B. cinerea* transcripts from healthy grape berries during the respective stages of NR as previously demonstrated by Otto et al. (2022). This is as the fungus begins to initiate the physicochemical changes associated with the NR process such as the degradation of the skin (Hegyi-Kaló et al., 2020), and the increase in the sugar content (Blanco-Ulate et al., 2015; Lovato et al., 2019; Otto et al., 2022). The observed increase in functional transcripts from NR to GR phase may be attribute due to the activation of *V. vinifera* defence responses, as previously demonstrated by Mehari et al. (2018) and Haile et al. (2017, 2020).

It is hypothesised that distinct grape genes are expressed in NR and GR berries. A comparable outcome was also observed by Pogány et al. (2022), who conducted a principal component analysis (PCA) on *V. vinifera* transcriptome genes with samples obtained from healthy berries, and berries showing early and late onset of NR respectively. The result of these analyses, indicate that the late NR phase was grouped separately from the other phases. The combined effect of multiple environmental variables including the influence of climate on the NR process has been previously demonstrated by Ribéreau-Gayon et al. (2006) and Blanco-Ulate et al. (2015). The ordination plot indicates that the GR condition and the DR conditions exhibit identical *Vitis vinifera* gene expression profiles. This is confirmed by the widely documented phenomenon that any preliminary phase of noble rot can evolve into grey rot under conditions of severe weather (Magyar, 2011). However, the observation that the genes expressed in NR samples are markedly distinct from both GR and DR genes represents a completely novel finding. These results provide a novel interpretation of the concept of noble and grey rot. It was previously assumed that a continuous specific environmental condition during rot is necessary for the process to lead to NR berries. However, when conditions change, GR occurred, which results in yield loss. In contrast, the initial stage of NR berry development is more akin to the GR phase, and it is only the mature NR phase that differs from GR in terms of grapevine gene expression, not the intermediate phases of the process. This does not negate the observation that why H, NR, DR and GR grape berries can present on the same grape bunch in the same vineyards at a given sampling time (Fournier et al., 2013). However, this provides a different interpretation of the current state of the berry and its potential for further development.

### 4.2 The identified metabolic pathways in different rot types

The majority of plant-pathogen interactions do not occur as a result of pathogen-induced plant immune responses or preformed plant defensive barriers. Rather they are expression of modulations in plant metabolism including amino acid, carbohydrate, and fatty acid metabolism, resulting in secondary metabolites associated with plant defence (Proels and Hückelhoven, 2014). A functional analysis was conducted on the annotated gene sets and k-means classification was employed to identify clusters within the gene sets corresponding to different rot types. The results obtained were consistent with those of the NMDS analysis which was conducted on the three berry types tested. In all three cases, a single prominent functional gene cluster was identified (**Figure 4**). These clusters for GR and DR are primarily associated with the plant’s response to pathogen infection, whereas the NR clusters are linked to functions that define the plant’s normal functioning in the absence of pathogen interaction.

For NR with the highest enrichment strength, we identified genes from the RNA polymerase pathway and L-lactate dehydrogenase (VVI00640) from the propanoate metabolism pathway. Lactate dehydrogenases are key enzymes in plant metabolism catalysing the conversion of 2-hydroxyacids to their corresponding 2-ketoacids and operate in the final stages of glucose metabolism (Chatterjee et al., 2023). The core cluster of NR from the valine, leucine and isoleucine degradation pathway (VVI280) was found to be enriched for genes. These amino acids are precursors of the corresponding 3-alkyl-2-methoxypyrazines, which act as an odour signal to deter potential predators, and effectively prevent vegetative tissues or unripe fruits from being eaten (Lei et al., 2018). From the oxidative phosphorylation pathway (VVI00190) we identified genes that encode the NAD(P)H-quinone oxidoreductase subunit 1, NAD(P)H-quinone oxidoreductase subunit 5, NADH dehydrogenase subunit 9 and cytochrome c oxidase subunit 2 coding genes. These correspond to enzymes that provide ATP to power cell functions. Two enzymes from the cysteine and methionine metabolism pathway are identified as enriched which represents a crucial process in the fruit ripening mechanism and in the response to heat stress (Wang et al., 2021). In conclusion, the characteristics of the NR core cluster properties, the enriched genes are related to general ripening processes with the absence of significantly enriched plant defence pathways. This is a noteworthy discovery for two reasons. Firstly, previous research has demonstrated that this berry state is biologically inactive and essentially dormant with regard to grapevine function (Elad et al., 2016). This claim is strongly refuted by the substantial number of functional genes that have been identified (**Figure 3**). Secondly, if this plant state is assumed to be alive, it does not exhibit any significant pathological response, despite the fact that this berry is typically completely colonised by *Botrytis cinerea* mycelia. Additionally, previous research has demonstrated that several fungi in addition to *B. cinerea* are actively metabolising these berries (Hegyi et al., 2023). Considering these observations, it can be posited that the grape in focus, which is heavily colonised by fungi, can be considered as inactive with respect to plant-pathogen interaction. This to say that the fungi have no discernible effect on the gene expression of the plant, no defence reactions are induced, and the condition can be considered as biologically quiescent. This may be the key to the long-term persistence of berries in this condition on the vine even the absence of significant weather fluctuations.

In case of DR and GR cases the core gene clusters of the transcriptome show significant overlap. This indicates which genes are most likely to be activated during plant stress responses and represent steps in the defence mechanisms against pathogens (AbuQamar et al., 2016). The Taurine and Hypotaurine Metabolism pathway (VVI00430) was found to be significantly enriched in both clusters in which glutamate decarboxylase was identified. Taurine or hypotaurine can regulate pectin and cell metabolism pathways, thereby conferring resistance to pathogen biodegradation (Liu et al., 2022). An isoquinoline alkaloid biosynthesis pathway (VVI00950) was identified as being enriched in genes encoding amine oxidase and aspartate aminotransferase. Isoquinoline has been demonstrated to exert a range of effects on biotic and abiotic stress factors, including antifungal, antiviral, antioxidant, and enzyme inhibitor activity (Dey et al., 2020). Furthermore, the tropane, piperidine and pyridine alkaloid biosynthesis pathway (VVI00960) was found to be significantly active in both NR and DR berries. This pathway plays an important role in heat, drought and combined abiotic stress responses (Ju et al., 2021). Additionally, the alanine, aspartate, and glutamate metabolism pathway (VVI00250) were identified as enriched in the two specific gene clusters. This pathway plays a crucial role in the ripening process of the fruit, particularly in the development of astringency-related sensory characteristics (Feng et al., 2024). Additionally, the phenylalanine metabolism pathway (VVI00360) was found to be enriched in both core clusters. This pathway has been demonstrated to enhance antioxidant activities by regulating phenolic biosynthesis (Manela et al., 2015). The identified pathways of arginine (VVI00220), tyrosine (VVI00350), and cyan amino acid metabolism (VVI00460) have been demonstrated to play a role in the suppression of pathogen-induced ROS levels (Lu et al., 2022; Huang et al., 2023 and Huang et al., 2020). In addition to the various pathways related to amino acid metabolism, genes representing plant-pathogen interaction (VVI04626) are members of the core cluster of GR but not of DR. Furthermore, the inclusion of non-clustered, significantly enriched genes in addition to the core cluster members allows for the identification of additional functions that play a role in pathogen-plant interactions and signalling mechanisms.

### 4.3 The function the major enriched genes of NR phase

The presence of multiple transcription-related genes found in the NR berries, including MYC (VIT_00013156001, VIT_00013156001), TGA (VIT_00036649001), WRKY (VIT_00024624001, VIT_00026965001, VIT_00024624001) and ERF1 (LOC100241302), can be primarily attributed to the transcriptional programming that occurs between DR and NR. Transcription factors facilitate the reprogramming between defence-associated genes and the biosynthesis of chemical metabolites (Howe et al., 2018). The identification of the WRKY transcription factor in the current study by Lovato et al. (2019); Haile et al. (2020), and Mehari et al. (2018) is not unexpected as it is a transcription factor commonly associated with grapevine (Wang et al., 2014) and has been shown to regulate both positive and negative defence responses. Several studies have demonstrated the key role of carbohydrates in plant defence responses to biotic and abiotic stress factors (Chen et al., 2010: Keunen et al., 2013). In addition to their role in cell wall modifications saccharides such as fructose, raffinose and trehalose enable metabolic sensing and signalling in plants (Morkunas et al., 2005). In the current study, a raffinose synthase (VIT_100242394) was identified which converts galactinol to raffinose (Li et al., 2020). Of particular interest here was the cell wall apoplastic invertase (VIT_00016869001), which was identified within the enriched galactose pathway in NR berries. This enzyme is of particular importance due to its ability to hydrolyses sucrose into glucose and fructose and thereby regulating carbon partitioning and sugar metabolism (Ruan et al., 2010). Additionally, this enzyme has also been implicated in several functions, including cellulose biosynthesis (Rende et al., 2017), suggesting a role in tolerance to abiotic stress.

### 4.4 The function of the major enriched genes of GR phase

The DR and GR transcriptomes share numerous non-clustering genes with high enrichment ratios, underscoring the importance of considering these pathways to gain a comprehensive understanding of the overall context. In addition to their role as being building blocks for protein synthesis, amino acids have been demonstrated to play an active role in plant development, response to environmental stress and defence (Trovato et al., 2021). Additionally, two amino-acid related enriched pathways, namely phenylalanine and tyrosine pathways were identified as being of particular relevance. A tyrosine aminotransferase (VIT_00014286001) was identified within these pathways, which is directly involved in the synthesis of l-phenylalanine and tyrosine respectively (Prabhu and Hudson, 2010). Furthermore, amidase (VIT_00000036001) was present in the enriched alanine and aspartate metabolism pathway which have been linked to plant growth and stress response (Moya-Cuevas et al., 2021). Glutamate metabolism (GM) plays a pivotal role in amino acid metabolism and regulates essential metabolic functions including plant defence (Seifi et al., 2013). For example, the enzyme glutathione S-transferase (GST) (VIT_00014973001) has been demonstrated to be responsible for the detoxification of herbicides such as, atrazine (Lamoureux et al., 1970). This enzyme was observed to be upregulated in healthy and GR berries (Mehari et al., 2018). Glutathione S-transferase enzymes have also been shown to possess glutathione peroxidase activities thereby implicating them in antioxidative defence responses (Dixon et al., 2002). Pogány et al. (2022) observed an upregulation of these enzymes in both NR and GR. Glutathione S-transferase catalyses the conjugation of electrophilic substrates to glutathione (Kural et al., 2019). The latter enzyme, in addition to a glutamine synthetase (VIT_00011072001) (Silva and Carvalho, 2013) and glutamate dehydrogenase (VIT_100232993) (Labboun et al., 2009) found in GR berries, plays a significant role in the recognition and signalling process associated with plant defence (Datta et al., 2015).

Fatty acids play an important role in plant defence activation, functioning as modulators and components of cellular membranes in glycolipids, as a reserve of energy and carbon in triacylglycerol (TAG), reservoirs of extracellular barrier components and regulators of stress signalling (He and Ding, 2020). In cases of GR and DR, the involvement of fatty acid biosynthetic enzymes has been demonstrated. These include beta-estradiol 17-dehydrogenase (LOC100244068), acyl-[acyl-carrier-protein] desaturase (VIT_00038175001, Hernández et al., 2019) and an acetyl-CoA acyltransferase 1 (VIT_00028557001, Wang et al., 2022), fatty acid elongation mitochondrial enoyl-acyl-carrier protein reductase (VIT_00025089001, Massengo-Tiassé and Cronan, 2009), a s-malonyltransferase (VIT_00024638001, Chen et al., 2017) and enoyl-acyl-carrier protein reductase I (LOC100241680, Heath and Rock, 1995) which were identified as belonging to enriched fatty acid biosynthesis pathways. Methyljasmonate and its free acid jasmonic acid (JA), which are synthetised via the enriched alpha-linoleic pathway, induce plant defences against a group of pathogens through recognition and signalling (Kazan and Manners, 2008). This process is facilitated by biosynthetic enzymes, including jasmonic acid-amido synthase (JAR1) and jasmonate (ZIM) domain-containing protein.

The identification of additional enzymes involved in phytohormone biosynthesis, including those associated with ethylene synthesis, such as an ethylene receptor-like protein (ETR) and an ethylene-responsive transcription factor 1B (ERF1/2 Wen et al., 2012), the following enzymes were identified: 1-aminocyclopropane-1-carboxylate synthase 1 (ACS6, VIT_00026962001, Polko and Kieber, 2019), ethylene receptor (ETR/ERS), ethylene-responsive transcription factor 1 (ERF1, Wen et al., 2012), basic endochitinase B (ChiB, VIT_00023922001, Gu et al., 2019), and auxin synthesis namely auxin-responsive protein IAA (AUX/AA, VIT_00009238001, Luo et al., 2018). It is unsurprising that auxin-responsive protein SAUR (SAUR, LOC100240974, Stortenbeker and Bemer, 2019) are present within the enriched plant hormone signal transduction pathway, given that they have been highlighted as being of crucial importance for the NR process (Blanco-Ulate et al., 2015; Lovato et al., 2019; Pogány et al., 2022).

The enriched pathways of DR and GR revealed the presence of genes that are responsive to osmotic stress including epidermal patterning factor 1 (EPF1/2, VIT_00014396001, Hara et al., 2007), dehydration (ERF transcription factor, LOC100241302), or drought, such as abscisic acid receptor (PYR/PYL, LOC100242938, Zhang et al., 2019), and wounding such as calmodulin (CaM4, VIT_00025831001, Peng et al., 2014), respiratory burst oxidase (RbohD, VIT_00014350001, Miller et al., 2009), protein epidermal patterning factor (EPF1/2, VIT_00014396001, Engineer et al., 2014) and cell wall strengthening calcium-dependent protein kinase (CDPK, VIT_00000238001, Fang et al., 2015), respiratory burst oxidase (RbohD, VIT_00014350001, Almagro et al., 2009) and calcium-binding protein CML (CaMCML, VIT_00004914001). Among these environmental stress related genes, several also have dual roles, influencing the redox status or reactive oxygen species (ROS) levels in *V. vinifera*. For example, the burst oxidase (RbohD), calmodulin (CaM4, Takahashi et al., 2011) and calcium-binding protein CML (CaMCML, McCormack et al., 2005) present in the enriched pathways of DR and GR or calcium-dependent protein kinase SK5 (CDPK, Boller and Felix, 2009) and cyclic nucleotide-gated channel (CNGC, LOC100241591) present in the enriched pathways of both rot types play a role in (PAMP)-immunity and thus oxidative stress. Moreover, the transcriptomes of DR and GR exhibited highest enrichment ratios with the identification of pathways such as phenylpropanoid and stilbene (Sharma et al., 2019), isoquinoline, taurine (Surai et al., 2021) and ubiquinone (Ácsová et al., 2021). Additionally, the enriched oxidative phosphorylation pathway was also identifying. These contribute to the non-enzymatic antioxidant pool thereby influencing the redox status of *V. vinifera* and consequently its defence status.

### 4.5 Significant differences between NR and GR, the functions of oppositely expressed genes

Significantly enriched pathways for GR upregulated and NR downregulated were identified as glycolysis, fructose and mannose metabolism, pentose phosphate pathway and carbon metabolism pathways. In each of these cases, genes are upregulated (ATP-dependent 6-phosphofructokinase, fructose-bisphosphate aldolase, phosphoglycerate kinase and mutase, and pyruvate kinase isozyme A) which serve as the initial step of aerobic respiration in the fructose-6-phosphate to pyruvate pathway. Within the general amino acid biosynthesis pathway, genes related to phenylalanine metabolism were found to be significantly enriched in NR berries. Furthermore, the phenylalanine metabolism pathway was also identified as being enriched. In this pathway, aspartate aminotransferase converts phenylalanine to phenylpyruvate, which serves as a precursor for the off-odour components phenylacetate and phenylacetic acid in wine (Campo et al., 2012). With regards to the galactose metabolism pathway, we identified genes that are upregulated and related to biosynthesis of raffinose and tagatose (raffinose synthase and 6-phosphofructokinase, respectively), the oligosaccharides that are involved in the cold-responsive stress response system (Sun et al., 2018).

The sulphur relay system yielded a high enrichment ratio was, and the gene encoding the enzyme cysteine desulfurase was identified as laying a pivotal role in various stages of the process. For instance, cysteine desulfurase may be involved in the production of specific H2S in different plant species. This process that is likely to be influenced by various growth and stress conditions, although these are largely unknown (Vojtovič et al., 2021). In the nicotinate and nicotinamide pathway, several key genes were identified as playing a role in the biosynthesis of nicotinate D-ribonucleoside (L-aspartate oxidase, nicotinate nucleotide pyrophosphorylase, and nicotinate phosphoribosyl transferase), a metabolite that has been proposed as a potential product of cell degradation by plant pathogens (Domingos et al., 2016 and Qu et al., 2022).

In the comparison of GR vs. NR, a significant enrichment of genes involved in the plant pathogen interaction process was also observed. These provide perhaps the insight into the fundamental differences between the two types of rot. The genes encoding calcium-dependent protein kinase (CDPK), WRKY transcription factor (WRKYTF), PTI1-like tyrosine protein kinase (Pti1), a putative disease resistance protein (RSP2), and the SGT1 homologue exhibited a significant increase in expression in GR while a decrease was observed in NR. These genes play a key role in the hypersensitive response, specifically in effector-triggered immunity (ETI) and pathogen-associated molecular pattern-triggered immunity (PAMP-TI or PTI) (Shang et al., 2006). Notably, genes that encoding the RAR1 and HSP90 proteins which are involved in the response are absent from the upregulated pathway. In the GR berry, genes that activate calcium signalling in response to fungal PAMP are upregulated, resulting in induction of defence-related genes. Additionally, the hypersensitive response system normally induced by pathogen secretion, and it has been reported that PTI and ETI require each other to confer robust disease resistance (Ngou et al., 2021).

**Figure 5** presents a summary of the genes that have been demonstrated to be significantly up-regulated in the plant-pathogen interaction pathway for the different types of rots. The most notable distinction is the contrast between the genes that trigger the hypersensitive response, which is SGT1 for GR and HSP90 for NR. SGT1 is a fungal or bacterial response factor, whereas HSP90 is known to be induced by heat stress. It is not possible to determine whether the response identified in our studies is induced by heat stress in the case of NR, as the sampling period was a moderate heat exposure for grapes in October. The observation that the pathogen-induced hypersensitive response was simplified for SGT1-downregulated NR (illustrated by the red box in **Figure 5**) suggests that the distinction between NR and GR is also reflected in the plant response mechanisms. The figure illustrates that a completely alternate pathway is engaged for NR in the context of plant-pathogen interaction, namely the flagellin-sensitive pathway. It is also noteworthy that the initial step of the calcium signalling pathway, cyanogenic glucosides (CNGs), plays a pivotal role in NR, whereas the subsequent portion of the pathway (calcium-dependent protein kinase - CDPK and calmodulin-like protein - Cam/CML) is active in GR and DR, with CDPK being downregulated in NR. Further investigation, with a specific focus on calcium signalling processes in noble and grey rot berries, is essential to gain a deeper understanding of this phenomenon.

The results the ANOVA tests indicate a decline in the defence response of *V. vinifera* during the NR phases, followed by an increase during the GR and DR phases. The significant decrease in the functional diversity of the *V. vinifera* gene expression profile across the DR and GR phases aligns with findings of Lovato et al. (2019), who underscore the significance of specific transcriptional reprogramming during the NR, including the downregulation of defence response related genes. The increase of defence related genes from the NR phase to GR is in consistence with the findings of Haile et al. (2017, 2020), who indicated that defence related genes continue to be activated during GR. However, this is futile, as *B. cinerea* exhibits a necrotrophic lifestyle during GR and expresses genes with phytotoxic activity, including botcinic acid and botrydial acid (Lovato et al., 2019; Otto et al., 2022) which cause plant damage by killing plant tissue. The increase in defence-related genes in infected plant tissues upon infection of plant host tissues by a phytopathogen is a common phenomenon (Alkan et al., 2015; Agudelo-Romero et al., 2015; Kelloniemi et al., 2015). This phenomenon may be attributed to two potential causes: futile attempts of the infected tissue to respond to the pathogen or the transcriptomic response of other cell layers that have not yet been colonised. The transition from an initial biotrophic to necrotrophic lifestyle or vice versa has previously been demonstrated for other plant pathogens, including *B. cinerea* (Glazebrook, 2005). This dual behaviour offers a wealth of valuable insight that can enhance the efficacy of both crop protection and viticultural technologies.

## 5 Conclusion

This study has provided a comprehensive and coherent overview of the differences in the gene expression profile of *V. vinifera* between noble rot and grey rot, with a particular focus on plant genes, and defence mechanisms that are key functions of the GR and DR phases. These results provide a novel interpretation of the plant response to noble rot and grey rot. The hypothesis was that a continuous specific environmental condition during the harvest period of *Botrytis*-infected grapes would result in the development of NR berries. However, when conditions changed, the development of GR berries, which is characterized by yield losses, would occur. In contrast, the initial stage of NR berry development closely resembles those of the GR phase. It is only at the completely developed NR phase that differs from GR in terms of grapevine gene expression, and not at the intermediate phases of the process. The similarity of these berry types indicates the potential for a transition between these phases, which could explain why grape berries from any stage of the DR phases can turn to GR and why all types of grape berries can be observed in the same field and sometimes even on the same grape bunch. Genes that were upregulated in GR and downregulated in NR showed activity in the plant pathogen interaction pathway, which is indicative of the activation of the plant response system and the hypersensitive response through the SGT1 protein. However, the RAR1 and HSP90 genes, which regulate a similar response, were not activated. This identified a potential stress-response pathway that is activated in the GR berry but not in the NR berry or in any other hypersensitive response activator. Conversely, in NR the gene expression profile demonstrates a highly diverse secondary metabolism related gene expression profile, but the pathogenic response reactions were less significant in this case. This difference gives a rise to a novel hypothesis, that, in contrast to our previous understanding that the developed NR berry is an inactive effectively necrotic berry, we found that several biological functions are active in the plant. However, these are not anti-pathogenic processes, but rather of a maturation nature. The comprehensive analysis of the defence mechanisms in *V. vinifera* reveals that the plant exhibits the most robust defence responses in GR berries. *V. vinifera* increases its defence responses, but these are ineffective, because during this phase, *B. cinerea* alters its virulence repertoire with the deployment of virulence factors that cause necrotrophic interaction. It can thus be conducted that the virulence expression profile of *B. cinerea* represents to be the critical factor in the onset of NR and GR respectively.

## Data availability statement

The datasets presented in this study can be found in online repositories. The names of the repository/repositories and accession number(s) can be found in the article/**Supplementary Material**.

## Author contributions

KV: Writing – original draft, Writing – review & editing, Conceptualization, Funding acquisition, Methodology, Supervision. MO: Data curation, Software, Writing – original draft. AG-T: Data curation, Investigation, Writing – original draft. AG: Data curation, Investigation, Writing – original draft. RG: Data curation, Investigation, Writing – original draft. JH-K: Data curation, Investigation, Methodology, Writing – original draft. TC: Data curation, Writing – original draft. JG: Conceptualization, Methodology, Validation, Writing – original draft. ZZ: Conceptualization, Methodology, Writing – review & editing. ÁH: Conceptualization, Data curation, Formal analysis, Methodology, Software, Writing – original draft.

## References

[B1] AbuQamar S. F. Moustafa K. Tran L. S. P. (2016). ‘Omics’ and plant responses to *Botrytis cinerea* . Front. Plant Sci. 7, 231838. doi: 10.3389/fpls.2016.01658 PMC5108755 27895649

[B2] Ácsová A. Hojerová J. Tobolková B. Martiniaková S. (2021). Antioxidant Efficacy of Natural Ubiquinol Compared to Synthetic References–In Vitro Study. ChemistrySelect. 6, 4495–4505.

[B3] Agudelo-Romero P. Erban A. Rego C. Carbonell-Bejerano P. Nascimento T. Sousa L.. (2015). Transcriptome and metabolome reprogramming in *Vitis vinifera* cv. Trincadeira berries upon infection with *Botrytis cinerea* . J. Exp. Bot. 66, 1769–1785. doi: 10.1093/jxb/eru517 25675955 PMC4669548

[B4] Alkan N. Friedlander G. Ment D. Prusky D. Fluhr R. (2015). Simultaneous transcriptome analysis of C olletotrichum gloeosporioides and tomato fruit pathosystem reveals novel fungal pathogenicity and fruit defense strategies. New Phytol. 205, 801–815. doi: 10.1111/nph.13087 25377514

[B5] Almagro L. Gómez Ros L. V. Belchi-Navarro S. Bru R. Ros Barceló A. Pedreño M. A. (2009). Class III peroxidases in plant defence reactions. J. Exp. Bot. 60, 377–390. doi: 10.1093/jxb/ern277 19073963

[B6] Blanco-Ulate B. Amrine K. C. Collins T. S. Rivero R. M. Vicente A. R. Morales-Cruz A.. (2015). Developmental and metabolic plasticity of white-skinned grape berries in response to *Botrytis cinerea* during noble rot. Plant Physiol. 169, 2422–2443. doi: 10.1104/pp.15.00852 26450706 PMC4677888

[B7] Boller T. Felix G. (2009). A renaissance of elicitors: perception of microbe-associated molecular patterns and danger signals by pattern-recognition receptors. Annu. Rev. Plant Biol. 60, 379–406. doi: 10.1146/annurev.arplant.57.032905.105346 19400727

[B8] Boutet E. Lieberherr D. Tognolli M. Schneider M. Bairoch A. (2007). “UniProtKB/Swiss-Prot: the manually annotated section of the UniProt KnowledgeBase,” in Plant bioinformatics: methods and protocols (Humana Press, Totowa, NJ), 89–112.

[B9] Campo E. Saenz-Navajas M. P. Cacho J. Ferreira V. (2012). Consumer rejection threshold of ethyl phenylacetate and phenylacetic acid, compounds responsible for the sweet-like off odour in wines made from sour rotten grapes. Aust. J. Grape Wine Res. 18, 280–286. doi: 10.1111/j.1755-0238.2012.00198.x

[B10] Chatterjee Y. Bhowal B. Gupta K. J. Pareek A. Singla-Pareek S. L. (2023). Lactate dehydrogenase superfamily in rice and *arabidopsis*: understanding the molecular evolution and structural diversity. Int. J. Mol. Sci. 24, 5900. doi: 10.3390/ijms24065900 36982973 PMC10057475

[B11] Chen J. W. Liu W. J. Hu D. X. Wang X. Balamurugan S. Alimujiang A.. (2017). Identification of a malonyl CoA-acyl carrier protein transacylase and its regulatory role in fatty acid biosynthesis in oleaginous microalga Nannochloropsis oceanica. Biotechnol. Appl. Biochem. 64, 620–626. doi: 10.1002/bab.1531 27572053

[B12] Chen L. Q. Hou B. H. Lalonde S. Takanaga H. Hartung M. L. Qu X. Q.. (2010). Sugar transporters for intercellular exchange and nutrition of pathogens. Nature 468, 527–532. doi: 10.1038/nature09606 21107422 PMC3000469

[B13] Datta R. Kumar D. Sultana A. Hazra S. Bhattacharyya D. Chattopadhyay S. (2015). Glutathione regulates 1-aminocyclopropane-1-carboxylate synthase transcription via WRKY33 and 1-aminocyclopropane-1-carboxylate oxidase by modulating messenger RNA stability to induce ethylene synthesis during stress. Plant Physiol. 169, 2963–2981. doi: 10.1104/pp.15.01543 26463088 PMC4677924

[B14] Dey P. Kundu A. Kumar A. Gupta M. Lee B. M. Bhakta T.. (2020). Analysis of alkaloids (indole alkaloids, isoquinoline alkaloids, tropane alkaloids). Recent Adv. Natural products Anal. pp, 505–567). doi: 10.1016/B978-0-12-816455-6.00015-9

[B15] Dixon R. A. Achnine L. Kota P. Liu C. J. Reddy M. S. Wang L. (2002). The phenylpropanoid pathway and plant defense—a genomics perspective. Mol. Plant Pathol. 3, 371–390. doi: 10.1046/j.1364-3703.2002.00131.x 20569344

[B16] Domingos S. Fino J. Paulo O. S. Oliveira C. M. Goulao L. F. (2016). Molecular candidates for early-stage flower-to-fruit transition in stenospermocarpic table grape (Vitis vinifera L.) inflorescences ascribed by differential transcriptome and metabolome profiles. Plant Sci. 244, 40–56. doi: 10.1016/j.plantsci.2015.12.009 26810452

[B17] Elad Y. Vivier M. Fillinger S. (2016). “ *Botrytis*, the good, the bad and the ugly,” in *Botrytis*–The fungus, the pathogen and its management in agricultural systems (Cham Swithzerland: Springer), 1–15.

[B18] Engineer C. B. Ghassemian M. Anderson J. C. Peck S. C. Hu H. Schroeder J. I. (2014). Carbonic anhydrases, EPF2 and a novel protease mediate CO2 control of stomatal development. Nature 513, 246–250. doi: 10.1038/nature13452 25043023 PMC4274335

[B19] Fang Y. Liao K. Du H. Xu Y. Song H. Li X.. (2015). A stress-responsive NAC transcription factor SNAC3 confers heat and drought tolerance through modulation of reactive oxygen species in rice. J. Exp. Bot. 66, 6803–6817. doi: 10.1093/jxb/erv386 26261267 PMC4623689

[B20] Feng J. Zhang W. Wang W. Nieuwenhuizen N. J. Atkinson R. G. Gao L.. (2024). Integrated transcriptomic and proteomic analysis identifies novel regulatory genes associated with plant growth regulator-induced astringency in grape berries. J. Agric. Food Chem. 72, 4433–4447. doi: 10.1021/acs.jafc.3c04408 38354220

[B21] Fournier E. Gladieux P. Giraud T. (2013). The ‘D r J ekyll and M r H yde fungus’: noble rot versus gray mold symptoms of *Botrytis cinerea* on grapes. Evolutionary Appl. 6, 960–969. doi: 10.1111/eva.12079 PMC3779096 24062804

[B22] Glazebrook J. (2005). Contrasting mechanisms of defense against biotrophic and necrotrophic pathogens. Annu. Rev. Phytopathol. 43, 205. doi: 10.1146/annurev.phyto.43.040204.135923 16078883

[B23] Gu S. Y. Wang L. C. Cheuh C. M. Lo W. S. (2019). CHITINASE like1 regulates root development of dark-grown seedlings by modulating ethylene biosynthesis in Arabidopsis thaliana. Front. Plant Sci. 10, 600. doi: 10.3389/fpls.2019.00600 31156671 PMC6530356

[B24] Haile Z. M. Malacarne G. Pilati S. Sonego P. Moretto M. Masuero D.. (2020). Dual transcriptome and metabolic analysis of *Vitis vinifera* cv. Pinot Noir berry and *Botrytis cinerea* during quiescence and egressed infection. Front. Plant Sci. 10, 1704. doi: 10.3389/fpls.2019.01704 32082332 PMC7002552

[B25] Haile Z. M. Pilati S. Sonego P. Malacarne G. Vrhovsek U. Engelen K.. (2017). Molecular analysis of the early interaction between the grapevine flower and Botrytis cinerea reveals that prompt activation of specific host pathways leads to fungus quiescence. Plant Cell Environ. 40, 1409–1428. doi: 10.1111/pce.12937 28239986

[B26] Hara K. Kajita R. Torii K. U. Bergmann D. C. Kakimoto T. (2007). The secretory peptide gene EPF1 enforces the stomatal one-cell-spacing rule. Genes Dev. 21, 1720–1725. doi: 10.1101/gad.1550707 17639078 PMC1920166

[B27] He M. Ding N. Z. (2020). Plant unsaturated fatty acids: multiple roles in stress response. Front. Plant Sci. 11, 562785. doi: 10.3389/fpls.2020.562785 33013981 PMC7500430

[B28] Heath R. J. Rock C. O. (1995). Enoyl-acyl carrier protein reductase (fabI) plays a determinant role in completing cycles of fatty acid elongation in Escherichia coli (∗). J. Biol. Chem. 270, 26538–26542. doi: 10.1074/jbc.270.44.26538 7592873

[B29] Hegyi Á.I. Otto M. Geml J. Hegyi-Kaló J. Geiger A. Golen R.. (2023). The origin of the particular aroma of noble rot wines: various fungi contribute to the development of the aroma profile of botrytised grape berries. OENO One 57, 165–176. doi: 10.20870/oeno-one.2023.57.3.7314

[B30] Hegyi A. I. Otto M. Geml J. Hegy-Kaló J. Kun J. Gyenesei A.. (2022). Metatranscriptomic analyses reveal the functional role of *Botrytis cinerea* in biochemical and textural changes during noble rot of grapevines. J. Fungi 8, 378. doi: 10.3390/jof8040378 PMC9030449 35448609

[B31] Hegyi-Kaló J. Hegyi A. I. Geml J. Zsófi Z. Pálfi X. Váczy K. Z. (2020). Physico-chemical characteristics and culturable microbial communities of grape berries change strongly during noble rot development. Plants 9, 1809. doi: 10.3390/plants9121809 33371257 PMC7766896

[B32] Hernández M. L. Sicardo M. D. Alfonso M. Martínez-Rivas J. M. (2019). Transcriptional regulation of stearoyl-acyl carrier protein desaturase genes in response to abiotic stresses leads to changes in the unsaturated fatty acids composition of olive mesocarp. Front. Plant Sci. 10, 251. doi: 10.3389/fpls.2019.00251 30891055 PMC6411816

[B33] Hornsey I. S. (2007). The chemistry and biology of winemaking (Cambridge, UK: Royal Society of Chemistry). doi: 10.1039/9781847557667

[B34] Howe A. C. Jansson J. K. Malfatti S. A. Tringe S. G. Tiedje J. M. Brown C. T. (2014). Tackling soil diversity with the assembly of large, complex metagenomes. Proceedings of the National Academy of Sciences, 111, 4904–4909. 10.1073/pnas.1402564111 PMC3977251 24632729

[B35] Howe G. A. Major I. T. Koo A. J. (2018). Modularity in jasmonate signaling for multistress resilience. Annu. Rev. Plant Biol. 69, 387–415. doi: 10.1146/annurev-arplant-042817-040047 29539269

[B36] Huang Y. Liang D. Xia H. Lin L. J. Wang J. Lv X. L. (2020). Lignin and quercetin synthesis underlies berry russeting in ‘sunshine muscat’grape. Biomolecules 10, 690. doi: 10.3390/biom10050690 32365571 PMC7277627

[B37] Huang X. Tang Q. Chen C. Li Q. Lin H. Bai S.. (2023). Combined analysis of transcriptome and metabolome provides insights into nano-selenium foliar applications to improve summer tea quality (*Camellia sinensis*). Lwt 175, 114496. doi: 10.1016/j.lwt.2023.114496

[B38] Ju Y. L. Min Z. Zhang Y. Zhang K. K. Liu M. Fang Y. L. (2021). Transcriptome profiling provide new insights into the molecular mechanism of grapevine response to heat, drought, and combined stress. Scientia Hortic. 286, 110076. doi: 10.1016/j.scienta.2021.110076

[B39] Kanehisa M. Furumichi M. Tanabe M. Sato Y. Morishima K. (2017). KEGG: new perspectives on genomes, pathways, diseases and drugs. Nucleic Acids Res. 45, D353–D361. doi: 10.1093/nar/gkw1092 27899662 PMC5210567

[B40] Kazan K. Manners J. M. (2008). Jasmonate signaling: toward an integrated view. Plant Physiol. 146, 1459–1468. doi: 10.1104/pp.107.115717 18390489 PMC2287326

[B41] Kelloniemi J. Trouvelot S. Héloir M. C. Simon A. Dalmais B. Frettinger P.. (2015). Analysis of the molecular dialogue between gray mold (*Botrytis cinerea*) and grapevine (*Vitis vinifera*) reveals a clear shift in defense mechanisms during berry ripening. Mol. Plant-Microbe Interact. 28, 1167–1180. doi: 10.1094/MPMI-02-15-0039-R 26267356

[B42] Keunen E. L. S. Peshev D. Vangronsveld J. Van Den Ende W. I. M. Cuypers A. N. N. (2013). Plant sugars are crucial players in the oxidative challenge during abiotic stress: extending the traditional concept. Plant Cell Environ. 36, 1242–1255. doi: 10.1111/pce.12061 23305614

[B43] Kural C. Kocdogan A. K. Şimşek G. G. Oğuztüzün S. Kaygın P. Yılmaz I.. (2019). Glutathione S-transferases and cytochrome P450 enzyme expression in patients with intracranial tumors: preliminary report of 55 patients. Med. Principles Pract. 28, 56–62. doi: 10.1159/000494496 PMC6558316 30321868

[B44] Labboun S. Tercé-Laforgue T. Roscher A. Bedu M. Restivo F. M. Velanis C. N.. (2009). Resolving the role of plant glutamate dehydrogenase. I. *in vivo* real time nuclear magnetic resonance spectroscopy experiments. Plant and cell physiology. 50, 1761–1773. 19690000 10.1093/pcp/pcp118 PMC2759343

[B45] Lamoureux G. L. Shimabukuro R. H. Swanson H. R. Frear D. S. (1970). Metabolism of 2-chloro-4-ethylamino-6-isopropylamino-s-triazine (atrazine) in excised sorghum leaf sections. J. Agric. Food Chem. 18, 81–86. doi: 10.1021/jf60167a029 5524468

[B46] Lei Y. Xie S. Guan X. Song C. Zhang Z. Meng J. (2018). Methoxypyrazines biosynthesis and metabolism in grape: A review. Food Chem. 245, 1141–1147. doi: 10.1016/j.foodchem.2017.11.056 29287333

[B47] Li T. Zhang Y. Liu Y. Li X. Hao G. Han Q.. (2020). Raffinose synthase enhances drought tolerance through raffinose synthesis or galactinol hydrolysis in maize and Arabidopsis plants. J. Biol. Chem. 295, 8064–8077. doi: 10.1074/jbc.RA120.013948 32366461 PMC7278351

[B48] Liu H. N. Pei M. S. Wei T. L. Yu Y. H. Guo D. L. (2022). ROS scavenger Hypotaurine delays postharvest softening of ‘Kyoho’grape by regulating pectin and cell metabolism pathway. Postharvest Biol. Technol. 186, 111833. doi: 10.1016/j.postharvbio.2022.111833

[B49] Lovato A. Zenoni S. Tornielli G. B. Colombo T. Vandelle E. Polverari A. (2019). Specific molecular interactions between *Vitis vinifera* and *Botrytis cinerea* are required for noble rot development in grape berries. Postharvest Biol. Technol. 156, 110924. doi: 10.1016/j.postharvbio.2019.05.025

[B50] Love M. I. Huber W. Anders S. (2014). Moderated estimation of fold change and dispersion for RNA-seq data with DESeq2. Genome Biol. 15, 1–21. doi: 10.1186/s13059-014-0550-8 PMC4302049 25516281

[B51] Lu X. Ma L. Zhang C. Yan H. Bao J. Gong M.. (2022). Grapevine (*Vitis vinifera*) responses to salt stress and alkali stress: transcriptional and metabolic profiling. BMC Plant Biol. 22, 528. doi: 10.1186/s12870-022-03907-z 36376811 PMC9661776

[B52] Luo J. Zhou J. J. Zhang J. Z. (2018). Aux/IAA gene family in plants: molecular structure, regulation, and function. Int. J. Mol. Sci. 19, 259. doi: 10.3390/ijms19010259 29337875 PMC5796205

[B53] Magyar I. (2006). Microbiological aspects of winemaking. PhD thesis. Corvinus University of Budapest, Faculty of Food Science, Budapest.

[B54] Magyar I. (2011). Botrytized wines. Adv. Food Nutr. Res. 63, 147–206. doi: 10.1016/B978-0-12-384927-4.00006-3 21867895

[B55] Manela N. Oliva M. Ovadia R. Sikron-Persi N. Ayenew B. Fait A.. (2015). Phenylalanine and tyrosine levels are rate-limiting factors in production of health promoting metabolites in *Vitis vinifera* cv. Gamay Red cell suspension. Front. Plant Sci. 6, 538. doi: 10.3389/fpls.2015.00538 26236327 PMC4503893

[B56] Massengo-Tiassé R. P. Cronan J. E. (2009). Diversity in enoyl-acyl carrier protein reductases. Cell. Mol. Life Sci. 66, 1507–1517. doi: 10.1007/s00018-009-8704-7 19151923 PMC2819910

[B57] McCormack E. Tsai Y. C. Braam J. (2005). Handling calcium signaling: arabidopsis CaMs and CMLs. Trends Plant Sci. 10, 383–389. doi: 10.1016/j.tplants.2005.07.001 16023399

[B58] Mehari Z. Malacarne G. Pilati S. Sonego P. Engelen K. Lionetti V.. (2018). The molecular dialogue between grapevine inflorescence/berry and *Botrytis cinerea* during initial, quiescent and egression infection stages. Acta Hortic. 1248, 587–594. doi: 10.17660/ActaHortic.2019.1248.79

[B59] Miller G. Schlauch K. Tam R. Cortes D. Torres M. A. Shulaev V.. (2009). The plant NADPH oxidase RBOHD mediates rapid systemic signaling in response to diverse stimuli. Sci. Signaling 2, ra45–ra45. doi: 10.1126/scisignal.2000448 19690331

[B60] Morales-Valle H. Silva L. C. Paterson R. R. M. Venâncio A. Lima N. (2011). Effects of the origins of *Botrytis cinerea* on earthy aromas from grape broth media further inoculated with *Penicillium expansum* . Food Microbiol. 28, 1048–1053. doi: 10.1016/j.fm.2011.02.005 21569951

[B61] Morkunas I. Marczak Ł. Stachowiak J. Stobiecki M. (2005). Sucrose-induced lupine defense against Fusarium oxysporum: Sucrose-stimulated accumulation of isoflavonoids as a defense response of lupine to Fusarium oxysporum. Plant Physiol. Biochem. 43, 363–373. doi: 10.1016/j.plaphy.2005.02.011 15907688

[B62] Moya-Cuevas J. Pérez-Alonso M. M. Ortiz-García P. Pollmann S. (2021). Beyond the usual suspects: physiological roles of the Arabidopsis amidase signature (AS) superfamily members in plant growth processes and stress responses. Biomolecules 11, 1207. doi: 10.3390/biom11081207 34439873 PMC8393822

[B63] Naegele R. P. (2018). Evaluation of host resistance to *Botrytis* bunch rot in Vitis spp. and its correlation with *Botrytis* leaf spot. HortScience 53, 204–207. doi: 10.21273/HORTSCI12655-17

[B64] Ngou B. P. M. Ahn H. K. Ding P. Jones J. D. (2021). Mutual potentiation of plant immunity by cell-surface and intracellular receptors. Nature 592, 110–115. doi: 10.1038/s41586-021-03315-7 33692545

[B65] Otto M. Geml J. Hegyi Á.I. Hegyi-Kaló J. Pierneef R. Pogány M.. (2022). *Botrytis cinerea* expression profile and metabolism differs between noble and grey rot of grapes. Food Microbiol. 106, 104037. doi: 10.1016/j.fm.2022.104037 35690441

[B66] Pell J. Hintze A. Canino-Koning R. Howe A. Tiedje J. M. Brown C. T. (2012). Scaling metagenome sequence assembly with probabilistic de Bruijn graphs. Proc. Natl. Acad. Sci. 109, 13272–13277. doi: 10.1073/pnas.1121464109 22847406 PMC3421212

[B67] Peng H. Yang T. Jurick W. M. (2014). Calmodulin gene expression in response to mechanical wounding and Botrytis cinerea infection in tomato fruit. Plants 3, 427–441. doi: 10.3390/plants3030427 27135512 PMC4844350

[B68] Pogány M. Dankó T. Hegyi-Kaló J. Kámán-Tóth E. Szám D. R. Hamow K.Á.. (2022). Redox and Hormonal Changes in the Transcriptome of Grape (*Vitis vinifera*) Berries during Natural Noble Rot Development. Plants 11, 864. doi: 10.3390/plants11070864 35406844 PMC9003472

[B69] Polko J. K. Kieber J. J. (2019). The regulation of cellulose biosynthesis in plants. Plant Cell 31, 282–296. doi: 10.1105/tpc.18.00760 30647077 PMC6447023

[B70] Prabhu P. R. Hudson A. O. (2010). Identification and partial characterization of an L-tyrosine aminotransferase (TAT) from Arabidopsis thaliana. Biochem. Res. Int. 2010, 11. doi: 10.1155/2010/549572 PMC3005984 21188077

[B71] Proels R. K. Hückelhoven R. (2014). Cell-wall invertases, key enzymes in the modulation of plant metabolism during defense responses. Mol. Plant Pathol. 15, 858–864. doi: 10.1111/mpp.12139 24646208 PMC6638650

[B72] Qu J. Z. Liu F. Pan X. X. Liao C. M. Li T. Zhang H. B.. (2022). Coordinative changes in metabolites in grape cells exposed to endophytic fungi and their extracts. Molecules 27, 5566. doi: 10.3390/molecules27175566 36080331 PMC9458220

[B73] R Core Team. (2023). _R: A Language and Environment for Statistical Computing_. R Foundation for Statistical Computing, Vienna, Austria. https://www.R-project.org/.

[B74] Reid K. E. Olsson N. Schlosser J. Peng F. Lund S. T. (2006). An optimized grapevine RNA isolation procedure and statistical determination of reference genes for real-time RT-PCR during berry development. BMC Plant Biol. 6, 1–11. doi: 10.1186/1471-2229-6-27 17105665 PMC1654153

[B75] Rende U. Wang W. Gandla M. L. Jönsson L. J. Niittylä T. (2017). Cytosolic invertase contributes to the supply of substrate for cellulose biosynthesis in developing wood. New Phytol. 214, 796–807. doi: 10.1111/nph.14392 28032636

[B76] Ribéreau-Gayon P. Dubourdieu D. Donèche B. Lonvaud A. (2006). Handbook of enology: The microbiology of wine and vinifications (Chicester: JohnWiley & Sons).

[B77] Ribéreau-Gayon J. Riberau-Gayon P. Seguin G. (1980). “ *Botrytis cinerea* in enology,” in The biology of botrytis. Eds. Coley-Smith J. R. Verhoeff K. Jarvis W. R. (Academic Press, London), 251–274.

[B78] Ruan Y. L. Jin Y. Yang Y. J. Li G. J. Boyer J. S. (2010). Sugar input, metabolism, and signaling mediated by invertase: roles in development, yield potential, and response to drought and heat. Mol. Plant 3, 942–955. doi: 10.1093/mp/ssq044 20729475

[B79] Seifi H. S. Van Bockhaven J. Angenon G. Höfte M. (2013). Glutamate metabolism in plant disease and defense: friend or foe? Mol. Plant-Microbe Interact. 26, 475–485. doi: 10.1094/MPMI-07-12-0176-CR 23342972

[B80] Shang Y. Li X. Cui H. He P. Thilmony R. Chintamanani S.. (2006). RAR1, a central player in plant immunity, is targeted by Pseudomonas syringae effector AvrB. Proc. Natl. Acad. Sci. 103, 19200–19205. doi: 10.1073/pnas.0607279103 17148606 PMC1748199

[B81] Sharma A. Shahzad B. Rehman A. Bhardwaj R. Landi M. Zheng B. (2019). Response of phenylpropanoid pathway and the role of polyphenols in plants under abiotic stress. Molecules 24, 2452. doi: 10.3390/molecules24132452 31277395 PMC6651195

[B82] Silva L. Carvalho H. (2013). Possible role of glutamine synthetase in the NO signaling response in root nodules by contributing to the antioxidant defenses. Front. Plant Sci. 4, 372. doi: 10.3389/fpls.2013.00372 24065976 PMC3777134

[B83] Stortenbeker N. Bemer M. (2019). The SAUR gene family: the plant’s toolbox for adaptation of growth and development. J. Exp. Bot. 70, 17–27. doi: 10.1093/jxb/ery332 30239806

[B84] Sun X. Matus J. T. Wong D. C. J. Wang Z. Chai F. Zhang L.. (2018). The GARP/MYB-related grape transcription factor AQUILO improves cold tolerance and promotes the accumulation of raffinose family oligosaccharides. J. Exp. Bot. 69, 1749–1764. doi: 10.1093/jxb/ery020 29385617 PMC5888914

[B85] Surai P. F. Earle-Payne K. Kidd M. T. (2021). Taurine as a natural antioxidant: From direct antioxidant effects to protective action in various toxicological models. Antioxidants 10, 1876. doi: 10.3390/antiox10121876 34942978 PMC8698923

[B86] Szklarczyk D. Kirsch R. Koutrouli M. Nastou K. Mehryary F. Hachilif R.. (2023). The STRING database in 2023: protein–protein association networks and functional enrichment analyses for any sequenced genome of interest. Nucleic Acids Res. 51, D638–D646. doi: 10.1093/nar/gkac1000 36370105 PMC9825434

[B87] Takahashi F. Mizoguchi T. Yoshida R. Ichimura K. Shinozaki K. (2011). Calmodulin-dependent activation of MAP kinase for ROS homeostasis in Arabidopsis. Mol. Cell 41, 649–660. doi: 10.1016/j.molcel.2011.02.029 21419340

[B88] Teissedre L. P. Donèche B. (2013). “Botrytized wines: sauternes, German wines,” in Sweet, reinforced and fortified wines: grape biochemistry, technology and vinification (Oxford, England: John Wiley & Sons), 285–299.

[B89] Thibon C. Cluzet S. Mérillon J. M. Darriet P. Dubourdieu D. (2011). 3-Sulfanylhexanol precursor biogenesis in grapevine cells: the stimulating effect of *Botrytis cinerea* . J. Agric. Food Chem. 59, 1344–1351. doi: 10.1021/jf103915y 21235257

[B90] Tominaga T. Niclass Y. Frérot E. Dubourdieu D. (2006). Stereoisomeric distribution of 3-mercaptohexan-1-ol and 3-mercaptohexyl acetate in dry and sweet white wines made from *Vitis vinifera* (Var. Sauvignon Blanc and Semillon). J. Agric. Food Chem. 54, 7251–7255. doi: 10.1021/jf061566v 16968090

[B91] Trovato M. Funck D. Forlani G. Okumoto S. Amir R. (2021). Amino acids in plants: regulation and functions in development and stress defense. Front. Plant Sci. 12. doi: 10.3389/978-2-88971-842-9 PMC8559698 34733310

[B92] Vannini A. Chilosi G. (2013). “Botrytis infection: grey mould and noble rot,” in Sweet, Reinforced and fortified wines: grape biochemistry, technology and vinification (Oxford, England: John Wiley & Sons), 159–169.

[B93] Vojtovič D. Luhová L. Petřivalský M. (2021). Something smells bad to plant pathogens: Production of hydrogen sulfide in plants and its role in plant defence responses. J. advanced Res. 27, 199–209. doi: 10.1016/j.jare.2020.09.005 PMC7728587 33318878

[B94] Wang W. Feng J. Wei L. Khalil-Ur-Rehman M. Nieuwenhuizen N. J. Yang L.. (2021). Transcriptomics integrated with free and bound terpenoid aroma profiling during “shine muscat”(Vitis labrusca× V. vinifera) grape berry development reveals coordinate regulation of MEP pathway and terpene synthase gene expression. J. Agric. Food Chem. 69, 1413–1429. doi: 10.1021/acs.jafc.0c06591 33481572

[B95] Wang M. Vannozzi A. Wang G. Liang Y. H. Tornielli G. B. Zenoni S.. (2014). Genome and transcriptome analysis of the grapevine (Vitis vinifera L.) WRKY gene family. Horticulture Res. 1, 16. doi: 10.1038/hortres.2014.16 PMC4596322 26504535

[B96] Wang M. Zheng Z. Tian Z. Zhang H. Zhu C. Yao X.. (2022). Molecular cloning and analysis of an acetyl-coA C-acetyltransferase gene (EkAACT) from euphorbia kansui liou. Plants 11, 1539. doi: 10.3390/plants11121539 35736690 PMC9229008

[B97] Wen X. Zhang C. Ji Y. Zhao Q. He W. An F.. (2012). Activation of ethylene signaling is mediated by nuclear translocation of the cleaved EIN2 carboxyl terminus. Cell Res. 22, 1613–1616. doi: 10.1038/cr.2012.145 23070300 PMC3494400

[B98] Williamson B. Tudzynski B. Tudzynski P. Van Kan J. A. (2007). Botrytis cinerea: the cause of grey mould disease. Mol. Plant Pathol. 8, 561–580. doi: 10.1111/j.1364-3703.2007.00417.x 20507522

[B99] Xie C. Mao X. Huang J. Ding Y. Wu J. Dong S.. (2011). KOBAS 2.0: a web server for annotation and identification of enriched pathways and diseases. Nucleic Acids Res. 39, W316–W322. doi: 10.1093/nar/gkr483 21715386 PMC3125809

[B100] Zenoni S. Fasoli M. Guzzo F. Dal Santo S. Amato A. Anesi A.. (2016). Disclosing the molecular basis of the postharvest life of berry in different grapevine genotypes. Plant Physiol. 172, 1821–1843. doi: 10.1104/pp.16.00865 27670818 PMC5100763

[B101] Zhang Q. Kong X. Yu Q. Ding Y. Li X. Yang Y. (2019). Responses of PYR/PYL/RCAR ABA receptors to contrasting stresses, heat and cold in Arabidopsis. Plant Signaling Behav. 14, 1670596. doi: 10.1080/15592324.2019.1670596 PMC6866694 31552801

