# Supplementary material for: Botrytis cinerea causes different plant responses in grape (Vitis vinifera) berries during noble and grey rot: diverse metabolism versus simple defence
Source: Front Plant Sci. 2024 Aug 6;15:1433161. doi: 10.3389/fpls.2024.1433161 (PMC11333459; doi:10.3389/fpls.2024.1433161)
Supplement: Supplementary file 1 [file Table_1.docx]

Supplementary Material

Supplementary table 1: Identified gene ontology terms for noble rot berry gene set.

| Term ID | Term description | False discovery rate (p-value) | |
| --- | --- | --- | --- |
| **Biological process** | | |  |
| GO:0008152 | Metabolic process | < 0.001 | |
| GO:0006012 | Galactose metabolic process | 0.0111 | |
| GO:0044281 | Small molecule metabolic process | 0.0111 | |
| GO:0044237 | Cellular metabolic process | 0.0157 | |
| GO:1901575 | Organic substance catabolic process | 0.0167 | |
| **Molecular function** | | |  |
| GO:0003824 | Catalytic activity | < 0.001 | |
| GO:0016491 | Oxidoreductase activity | < 0.001 | |
| GO:0015453 | Oxidoreduction-driven active transmembrane transporter activity | < 0.001 | |
| GO:0016651 | Oxidoreductase activity, acting on NAD(P)H | 0.0045 | |
| GO:0008137 | NADH dehydrogenase (ubiquinone) activity | 0.0082 | |
| GO:0009055 | Electron transfer activity | 0.0082 | |
| GO:0015399 | Primary active transmembrane transporter activity | 0.0146 | |
| GO:0046914 | Transition metal ion binding | 0.0146 | |
| GO:0047216 | Inositol 3-alpha-galactosyltransferase activity | 0.0146 | |
| GO:0103075 | indole-3-pyruvate monooxygenase activity | 0.0146 | |
| GO:0016616 | Oxidoreductase activity, acting on the CH-OH group of donors, NAD or NADP as acceptor | 0.0232 | |
| GO:0008270 | Zinc ion binding | 0.0439 | |

Supplementary table 2: Identified gene ontology terms for grey rot berry gene set.

| Term ID | Term description | False discovery rate (p-value) |
| --- | --- | --- |
| **Biological process** | | |
| GO:0006749 | Glutathione metabolic process | < 0.001 |
| GO:0006790 | Sulfur compound metabolic process | < 0.001 |
| GO:0006536 | Glutamate metabolic process | 0.0114 |
| GO:0006103 | 2-oxoglutarate metabolic process | 0.0392 |
| GO:0006531 | Aspartate metabolic process | 0.0392 |
| GO:0042221 | Response to chemical | 0.0392 |
| GO:1901564 | Organonitrogen compound metabolic process | 0.0392 |
| **Molecular function** | | |
| GO:0019842 | Vitamin binding | < 0.001 |
| GO:0004364 | Glutathione transferase activity | < 0.001 |
| GO:0030170 | Pyridoxal phosphate binding | < 0.001 |
| GO:0005509 | Calcium ion binding | < 0.001 |
| GO:0043167 | Ion binding | < 0.001 |
| GO:0031418 | L-ascorbic acid binding | 0.0013 |
| GO:0031406 | Carboxylic acid binding | 0.0019 |
| GO:0043177 | Organic acid binding | 0.0019 |
| GO:0003824 | Catalytic activity | 0.0027 |
| GO:0046872 | Metal ion binding | 0.0027 |
| GO:0004656 | Procollagen-proline 4-dioxygenase activity | 0.0158 |
| GO:0008242 | Omega peptidase activity | 0.0158 |
| GO:0070003 | Threonine-type peptidase activity | 0.0163 |
| GO:0004069 | L-aspartate:2-oxoglutarate aminotransferase activity | 0.0231 |
| GO:0008483 | Transaminase activity | 0.0265 |
| GO:0004427 | Inorganic diphosphatase activity | 0.0311 |
| GO:0016881 | Acid-amino acid ligase activity | 0.0328 |
| GO:0030246 | Carbohydrate binding | 0.0398 |
| GO:0043168 | Anion binding | 0.0398 |
| GO:0016641 | Oxidoreductase activity, acting on the CH-NH2 group of donors, oxygen as acceptor | 0.0477 |

Supplementary table 3: Identified gene ontology terms for developing rot berry gene set.

| Term ID | Term description | False discovery rate (p-value) |
| --- | --- | --- |
| **Biological process** | | |
| GO:0006749 | Glutathione metabolic process | < 0.001 |
| GO:0006790 | Sulfur compound metabolic process | 0.0011 |
| GO:0044281 | Small molecule metabolic process | 0.0034 |
| GO:1901564 | Organonitrogen compound metabolic process | 0.0034 |
| GO:0006536 | Glutamate metabolic process | 0.0076 |
| GO:0006807 | Nitrogen compound metabolic process | 0.0086 |
| GO:0008152 | Metabolic process | 0.0201 |
| GO:0006103 | 2-oxoglutarate metabolic process | 0.0230 |
| GO:0006520 | Cellular amino acid metabolic process | 0.0266 |
| GO:0006531 | Aspartate metabolic process | 0.0266 |
| GO:0071704 | Organic substance metabolic process | 0.0266 |
| GO:0009066 | Aspartate family amino acid metabolic process | 0.0269 |
| GO:0019752 | Carboxylic acid metabolic process | 0.0269 |
| GO:1901605 | Alpha-amino acid metabolic process | 0.0289 |
| GO:0005975 | Carbohydrate metabolic process | 0.0304 |
| GO:0018401 | Peptidyl-proline hydroxylation to 4-hydroxy-L-proline | 0.0304 |
| **Molecular function** | | |
| GO:0019842 | Vitamin binding | < 0.001 |
| GO:0003824 | Catalytic activity | < 0.001 |
| GO:0030170 | Pyridoxal phosphate binding | < 0.001 |
| GO:0004364 | Glutathione transferase activity | < 0.001 |
| GO:0004656 | Procollagen-proline 4-dioxygenase activity | 0.0338 |
| GO:0008242 | Omega peptidase activity | 0.0338 |
| GO:0043168 | Anion binding | 0.0338 |
| GO:0070003 | Threonine-type peptidase activity | 0.0338 |
| GO:0004069 | L-aspartate:2-oxoglutarate aminotransferase activity | 0.0346 |
| GO:0008483 | Transaminase activity | 0.0346 |
| GO:0016740 | Transferase activity | 0.0346 |
| GO:0031406 | Carboxylic acid binding | 0.0346 |
| GO:0043177 | Organic acid binding | 0.0346 |
| GO:0004427 | Inorganic diphosphatase activity | 0.0373 |
| GO:0016881 | Acid-amino acid ligase activity | 0.0390 |

Supplementary table 4: The gene names of the k-mean clustered functional networks for NR, DR and GR and the corresponding transcribed UniprotKB IDs.

| Gene name using KEGG nomenclature | UniprotKB protein ID |
| --- | --- |
| **Noble rot (NR)** | |
| 2-oxoisovalerate dehydrogenase | D7U4C7 |
| Alcohol dehydrogenase | E0CRP3, D7THH8 |
| Aldose 1-epimerase | D7SIE1 |
| Beta-amyrin synthase | F6GYJ2 |
| Branched-chain-amino-acid aminotransferase | E0CNW7 |
| Cell division cycle cofactor | A5BP73 |
| Chorismate synthase | D7SKX5 |
| Cytochrome c oxidase | D7UAQ1, B6VJY8 |
| dCMP deaminase | D7SGY9 |
| dCTP diphosphatase | F6H8R5 |
| Galactinol synthase | F6I4U8, D7UA62 |
| Glycerol-3-phosphate dehydrogenase | D7SPY8 |
| Indole-3-pyruvate monooxygenase | F6HZT7, F6GZU5 |
| L-lactate dehydrogenase | F6I382 |
| NADH dehydrogenase | Q0ZIX1, Q0ZIW4, B6VJV5 |
| Pectin esterase | F6HGZ1 |
| Peptidyl serine alpha-galactosyltransferase | D7SPX0 |
| RNA polymerase | Q0ZJ29, Q0ZJ30 |
| Stem adenine O-acetyltransferase | D7TAL3 |
| Trans-resveratrol di-O-methyltransferase | F6H5H8 |
| Ubiquitin-protein ligase | F6H779 |
| V-type proton ATPase | D7TVB7 |
| **Developing rot (DR)** | |
| 1-aminocyclopropane-1-carboxylate synthase | D7TV63 |
| Abscisic acid receptor | D7STN1 |
| Amine oxidase | D7T6W4 |
| Aspartate aminotransferase | D7TBL7, D7SR19 |
| Auxin responsive GH3 gene | F6HSW4, F6H697 |
| Auxin-responsive protein | A5C137 |
| Caffeic acid methyltransferase | F6H0K8 |
| Chitinase | D7T541 |
| CoA ligase | F6H0C0 |
| Endochitinase | F6HB09 |
| Ethylene-response factor | F6HVH5, F6H8M0 |
| Fructokinase-4 | A5B8T3 |
| Gluconokinase | D7T7U3 |
| Glutamate decarboxylase | F6HF90 |
| Glutathione hydrolase | D7TBD5 |
| Glutathione S-transferase | D7T7H2, F6GUW5, F6HJH8, A4LAG9, F6HR78 |
| Inorganic pyrophosphatase | D7U028 |
| L-asparaginase | A5C3M5 |
| Mannose-6-phosphate isomerase | F6HSR9 |
| NAD+ kinase | F6GU32 |
| Nitril hydratase | D7U3W2 |
| Phosphoserine aminotransferase | F6H8F3 |
| Prolyl-4-hydroxylase | E0CQW5, D7TWX6 |
| Pyridoxine 5-phosphate oxidase | D7U027 |
| Pyruvate kinase | F6HVY1 |
| Threonine synthase | F6HGV4 |
| TIFY 10A | F6HGN3 |
| Transcription factor TGA | F6HLL2 |
| Uridine nucleosidase | D7TJ39 |
| WRKY transcription factor | D7UCE2 |
| Xyloglucan endotransglucosylase | F6GXE0, F6GXE6 |
| **Grey rot (GR)** | |
| 1-aminocyclopropane-1-carboxylate synthase | D7TV63 |
| Abscisic acid receptor | D7STN1 |
| Allene oxide synthase | F6HQI7 |
| Amine oxidase | D7T6W4 |
| Aminocyclopropane-1-carboxylic acid oxidase | F6HIF0 |
| Aspartate aminotransferase | D7SR19, D7TBL7 |
| Auxin-responsive protein | A5C137 |
| Bifunctional nitrilase | D7U3W2 |
| Calcium binding protein | F6HG77, F6HG70, F6HG68, C5DB59, F6I6Y2, F6HG72 |
| CoA ligase | F6H0C0 |
| Cytochrome P450 | F6HSX3 |
| Ethylene-response factor | F6H8M0 |
| Glutamate decarboxylase | F6HF90 |
| Glutathione hydrolase | D7TBD5 |
| Glutathione S-transferase | F6GUW5, F6HJH8, F6HR78, D7T7H2, A4LAG9 |
| Indole-3-acetic acid amino synthetase | F6HSW4, F6H697 |
| Inorganic pyrophosphatase | D7U028 |
| L-asparaginase | A5C3M5 |
| NAD+ kinase | F6GU32 |
| P-type Cu+ transporter | A5B663 |
| Phosphoserine aminotransferase | F6H8F3 |
| Prolyl 4-hydroxylase | E0CQW5, D7TWX6 |
| Pyridoxine 5-phosphate synthase | D7U027 |
| Pyruvate kinase | F6HVY1 |
| Transcription factor TGA10 | F6HLL2 |
| Uridine nucleosidase | D7TJ39 |
| WRKY transcription factor | D7UCE2 |
| Xyloglucan endoglycosidase | F6GXE6 |
